# In vivo profiling of the endothelium using ‘AngioTag’ zebrafish

**DOI:** 10.1007/s10456-025-09990-8

**Published:** 2025-07-04

**Authors:** Mayumi F. Miller, Leah J. Greenspan, Derek E. Gildea, Kathryn Monzo, Gennady Margolin, Van N. Pham, Keith K. Ameyaw, Lisa Price, Natalie Aloi, Amber N. Stratman, Andrew E. Davis, Isabella Cisneros, Caleb A. Mertus, Ryan K. Dale, Andreas D. Baxevanis, Brant M. Weinstein

**Affiliations:** 1 Division of Developmental Biology, Eunice Kennedy Shriver National Institute of Child Health and Human Development, National Institutes of Health, Bethesda, MD 20892 USA; 2 Center for Genomics and Data Science Research, Division of Intramural Research, National Human Genome Research Institute, National Institutes of Health, Bethesda, MD 20892 USA; 3 Bioinformatics and Scientific Programming Core, Eunice Kennedy Shriver National Institute of Child Health and Human Development, National Institutes of Health, Bethesda, MD 20892 USA; 4 Present Address: Division of Applied Veterinary Research, Office of Applied Research, Center for Veterinary Medicine, Food and Drug Administration, Laurel, MD 20708 USA; 5 Present Address: Department of Chemical and Biological Sciences, Montgomery College, Takoma Park, MD USA; 6 Present Address: Department of Cell Biology and Physiology, Washington University School of Medicine, St. Louis, MO USA

**Keywords:** RiboTag, Translatome, TRAP-RNAseq, Endothelial cell profiling, Zebrafish

## Abstract

Vascular endothelial cells in vivo are exquisitely regulated by their local environment, which is disrupted or absent when using methods such as FACS sorting of cells isolated from animals or in vitro cell culture. Here, we profile the gene expression patterns of undisturbed endothelial cells in living animals using a novel “AngioTag” zebrafish transgenic line that permits isolation of actively translating mRNAs from endothelial cells in their native environment. This transgenic line uses the endothelial cell-specific *kdrl* promoter to drive expression of an epitope tagged Rpl10a 60 S ribosomal subunit protein, allowing for Translating Ribosome Affinity Purification (TRAP) of actively translating endothelial cell mRNAs. By performing TRAP-RNAseq on AngioTag animals, we demonstrate strong enrichment of endothelial-specific genes and have uncovered both novel endothelial genes and unique endothelial gene expression signatures for different adult organs. Finally, we generated a versatile “UAS: RiboTag” transgenic line to allow a wider array of different zebrafish cell and tissue types to be examined using TRAP-RNAseq methods. These new tools offer an unparalleled resource to study the molecular identity of cells in their normal in vivo context.

**Supplementary Information:**

The online version contains supplementary material available at 10.1007/s10456-025-09990-8.

## Introduction

The vascular system is a complex network of arteries, veins, and capillaries working in concert to allow oxygenated blood to flow throughout the body, transporting hormones and small molecules to target tissues. This network is comprised of lumenized endothelial cell tubes surrounded by smooth muscle cells (in larger vessels, especially arteries) or pericytes juxtaposed to the basement membrane. Endothelial cells in vascular tubes have extensive cell–cell junctional contacts with one another and with the surrounding vascular smooth muscle cells. In addition, the luminal surface of the endothelium is constantly exposed to flow dynamics, circulating cells, and circulating factors from the blood, while the abluminal surface interacts extensively with extracellular matrix and with numerous non-vascular cell types and tissues. Understanding how endothelial cells respond to this complex multitude of external cues and signals in vivo to regulate blood vessel growth and function is of enormous interest given the important roles vessels play in a variety of pathologies including cancer, ischemia, and congenital vascular disorders.

Existing methods for analysis of endothelial gene expression and function generally rely on post-mortem single-cell isolation and/or in vitro culture of endothelial cells, disrupting virtually all of the normal interactions experienced by endothelial cells in vivo*.* A number of animal models have been used to study endothelial cells in their endogenous setting, including mice, birds, and zebrafish. The zebrafish is a particularly useful model organism for studying the vasculature, with externally developing, optically clear embryos and larvae, as well as the availability of a variety of transgenic reporter lines expressing fluorescent proteins in the endothelium that permit high-resolution visualization of the complex and widely dispersed vascular network at all stages of development including adults [1, 2]. These features, along with the ability to house large numbers of adult animals and obtain large numbers of progeny, facilitate genetic and experimental analysis of vascular development and homeostasis in the fish. The advantages of the zebrafish model have led to important discoveries including mechanisms of arterial-venous differentiation [3–6], lumen formation [7–9], and guidance and patterning of vessel networks during development [10, 11].

Although zebrafish have been an invaluable model for observing endothelial cell behavior in vivo*,* existing methods for global analysis of endothelial gene expression, such as RNAseq or single-cell RNAseq approaches, still require enzymatic and mechanical dissociation into single-cell suspensions that are then subjected to further (and often lengthy) sorting procedures before RNA can be prepared from the cells. It is likely that these manipulations introduce signaling pathway changes, including activation of cell stress and apoptotic pathways, that obscure the native transcriptional state of endothelial cells. In contrast, Translating Ribosome Affinity Purification (TRAP) [12] involves collecting actively translating mRNAs by immunoprecipitating the attached ribosomes translating these mRNAs. Initial studies in mice have demonstrated that transgene-driven expression of an epitope-tagged ribosomal protein subunit (also known as a “RiboTag”) within specific cell types or tissues can be used to affinity purify and profile actively translating mRNAs in vivo by performing TRAP followed by RNAseq (TRAP-RNAseq) or microarray experiments [12–14]. These plus several studies in varying model organisms such as mice, zebrafish, and flies, have not only showed the versatility of this method in probing in vivo gene expression of different cell types, but have also thoroughly validated this tool by demonstrating the incorporation of tagged ribosomal subunits into the ribosome [12, 15–17] and the correlation of ribosome-associated mRNA transcripts with protein expression [12, 18–20].

We have now developed transgenic zebrafish that permit profiling of global gene expression in vascular endothelial cells (“AngioTag”) or in any cell or tissue type for which a Gal4 driver line is available (“UAS:RiboTag”). These lines utilize the vascular endothelial kdrl or UAS promoters, respectively, to drive co-expression of HA-tagged Rpl10a (a 60S ribosomal subunit protein) and EGFP (a visible transgene marker), permitting immunoprecipitation of tissue-specific ribosomes and collection of the mRNAs they are attached to and translating. Since isolation of vascular mRNA from these transgenic lines is extremely rapid, embryos and organs are quickly homogenized in a lysis buffer containing cycloheximide to halt further translation so that the collected mRNAs represent the *bona fide* expression profiles of undisrupted cells in situ*.* Using this tool, we have uncovered novel endothelial genes involved in embryonic vessel development, as well as unique endothelial signatures of vessels in different adult organs. These new transgenic lines provide powerful tools for profiling cell- or tissue-specific gene expression in the zebrafish that can be used to study changes in gene expression during development, disease, or repair after injury.

## Methods

### Zebrafish

Zebrafish were maintained and zebrafish experiments were performed according to standard protocols [21] and in conformity with the Guide for the Care and Use of Laboratory Animals of the National Institutes of Health, in an Association for Assessment and Accreditation of Laboratory Animal Care (AAALAC)-accredited facility. Fish were housed in a large recirculating aquaculture facility with 1.8 L and 6 L tanks. Water quality was routinely measured and proper parameters taken to maintain water quality stability. Fry were fed rotifers and adults were fed Gemma Micro 300 (Skretting). The following transgenic fish lines were used in this study: EK (wild-type), *Tg(kdrl:gfp)*^*la116*^ [22], *Tg(kdrl:egfp-2a-rpl10a3xHA)*^*y530*^ (this paper), *Tg(uas:egfp-2a-rpl10a2xHA)*^*y531*^ (this paper), *Tg(xa210:gal4)*^*y241*^ [23], *Tg(fli1a:gal4ff)*^*ubs4*^ [24], and *Tg(huc:gal4)* [25].

### Sucrose gradient

Sucrose density gradients were prepared for sedimentation analysis of polysome profiles. 12 mL 5–50% sucrose gradients were prepared in 110 mM KOAc, 2M MgOAc, 10 mM HEPES pH 7.6 with BioComp Gradient Master (BioComp), and allowed to rest overnight at 4 °C. Dechorionated and deyolked embryos were dounce homogenized in 1.5 µl polysome fractionation buffer per embryo (10mM HEPES pH7.4, 110mM KOAc, 2mM MgOAc, 100mM KCl, 10mM MgCl_2_, 0.1% Nonidet P-40, 2mM DTT, 40U/mL RNasin, 200ug/mL cycloheximide, and protease inhibitors; adapted from [26]). Homogenates were centrifuged at 1000xg for 10 min at 4 °C. Protein concentration was quantified by Bradford assay (Sigma Aldrich) and equivalent amounts were loaded onto gradients. Samples were centrifuged in a SW41 Beckman rotor at 40,000 rpm at 4 °C for 2 h. 16 × 1 mL fractions were collected with an ISCO piercing apparatus connected to a BioLogic chromatography system at 0.5 mL/min, pushing 55% sucrose. Data were collected using LP DataView software (BioRad). For EDTA treatment, samples were treated with 200 mM final concentration EDTA prior to loading onto gradient.

### TRAP protocol

Translating Ribosome Affinity Purification (TRAP) was performed as described previously [12] with modifications. For each larval TRAP sample, approximately 1200-1500 24 hpf zebrafish embryos were dechorionated, deyolked, and dounce homogenized in homogenization buffer consisting of 50 mM Tris pH 7.4, 100 mM KCl, 12 mM MgCl_2_, 1% NP-40, 1 mM DTT (Sigma, Cat. #646563), 1 × Protease inhibitors (Sigma, Cat. #P8340), 200 units/mL RNAsin (Promega, Cat. #N2115), 100ug/mL Cycloheximide (Sigma Cat. #7698), 1mg/mL Heparin (Sigma, Cat. #H3393-10KU). Lysates were cleared for 10 min at 10,000×*g*, 4 °C. 1 µl of anti-HA antibody (Abcam ab9110 Rabbit polyclonal, RRID: AB_307019) was added per 400 µg protein in an 800 µl lysate and the samples were orbitally rotated at 4 °C for 5 h. 60 µl of Dynabeads® protein G slurry (Novex/Life Technologies) was added per 1 µl of anti-HA antibody with homogenization. Before using, Dynabeads were washed with 800 µl homogenization buffer, rotating for 30 min to equilibrate the beads. The wash buffer was then removed from the Dynabeads and the lysate + Ab solution was added to the equilibrated Dynabeads. This Dynabead + lysate + Ab mixture was incubated overnight at 4 °C on an orbital rotator. Dynabeads were collected using a magnetic stand and washed 3 × 5 min on an orbital shaker with high salt homogenization buffer (50 mM Tris pH 7.4, 300 mM KCl, 12 mM MgCl_2_, 1% NP-40, 1 mM DTT, 1 × Protease inhibitors, 200 units/mL RNAsin, 100 µg/mL cyclohexamide, 1mg/mL heparin). RNA was collected from the Dynabeads and DNase treated using the Zymo ZR-Duet Kit (Zymo Research D7003). Note that careful attention to the protocol is required to keep lysates cold and free of RNases as RNA integrity must be carefully monitored. Large numbers of embryos are needed to provide the starting material for the TRAP protocol because (i) the endothelial or other cell types expressing the RiboTag represent a small fraction of the total cells in the animal, (ii) only a fraction of the ribosomes in the targeted cell type contain the RiboTag, with most ribosomes still being “untagged”; (iii) TRAP-purified RNA samples contain a large amount of co-purifying ribosomal RNAs in addition to the mRNAs, and (iv) we prepared largely unamplified libraries for sequencing.

A similar TRAP protocol was utilized for the adult whole fish and organ samples as described above with a few additional modifications. SUPERase-In (100 U/µl) was added to the homogenization buffer as an additional RNase inhibitor. Organs were either dissected fresh or flash frozen for later use. Whole fish were flash frozen and ground into fish powder before homogenization. Four organs/whole fish were used per replicate and the amount of each tissue was weighed so that a ratio of only 0.75–3% tissue to homogenization buffer (weight/volume) was utilized. Tissue was homogenized using a Cole-Parmer LabGen 850 homogenizer at 13,000 rpm for 45 s. 5 µl of anti-HA antibody was used per sample. Antibody and Dynabeads incubation were the same as the larval samples, but RNA was collected from the Dynabeads using RNeasy Micro Plus Kit (Qiagen #74034) with RNA-only β-mercaptoethanol supplementation in the RLT buffer.

### Quantitative RT-PCR

RNA was reverse transcribed using a high-capacity cDNA reverse transcription kit (Applied Biosystems™). cDNA was combined with the primers listed below and with LightCycler® 480 SYBR Green I (Roche). Reactions were run in a LightCycler® 480 Multiwell Plate 384 machine (Roche). Relative fold changes were calculated using ddCT calculations and by calculating standard deviations. The following primer pairs were used:

**Table:** 

Gene	Forward Primer	Reverse Primer
cdh5	CAACAGACGCTGATGATTCC	GTCTTTGGCTTGAACAGCAA
kdrl	GAGTTCCAGCACCCTTTATCA	ATCGTCCTTCTTCACCCTTTC
desmin a	CGGTGGTTATCAGGACACTATC	TCCAGAGCCATCTTCACATTC
neurogenin 1	CGCATTGGATGCTTTGAGAAG	CGAAAGTGCCCAGATGTAGT
snap25	GGCTACTGTCATGCTCCTTATT	TGATTGTAAGTGCTCGTCGTATTA
actin b1	CGAGCAGGAGATGGGAACC	CAACGGAAACGCTCATTGC

### RNAseq

RNA concentration and quality were measured on a Qubit (Thermo Fisher) and on an Agilent 2100 BioAnalyzer + RNA Pico Chip (Agilent), respectively. For our 24 hpf TRAP samples, a large proportion of the RNA samples we collected represented ribosomal RNAs co-purified with our polysome mRNAs, so 500 ng RNA was polyA-selected or rRNA-depleted using an Ribo-Zero rRNA Removal Kit (Illumina) before library construction. Sequencing libraries were constructed from the purified mRNA using TruSeq Stranded mRNA Library Prep Kits (Illumina). Libraries were sequenced using an Illumina HiSeq 2500 to generate approximately 50 million 2 × 75 bp or 2 × 100 bp pair end reads. Raw data were de-multiplexed and analyzed. RNAseq data were deposited into NCBI’s Gene Expression Omnibus (GEO) repository and assigned accession number GSE292080.

For adult whole fish and organs, RNA samples were submitted to Novogene, Inc. for ultralow input mRNA sequencing. Samples underwent quality control, library construction, and sequencing. RNA libraries for RNAseq were prepared by Novogene using a SMART-seq V4 Ultra Low Input RNA kit and a non-directional library. Messenger RNA was enriched using a poly-T oligo-attached magnetic beads. Samples were sequenced on an Illumina NovaSeq 6000 to generate 150 bp pair end reads ranging from 24 to 94 million raw reads per sample. RNAseq data were deposited into GEO and assigned accession number GSE276280.

### Assessing mRNA abundance from RNAseq data

For our larval TRAP and FACS samples, raw RNAseq reads were mapped to the zebrafish reference genome (GRCz11) using the STAR alignment software package (v. 2.7.11b). Only those RNAseq reads mapping to annotated protein-coding Ensembl gene models (release 99, https://ftp.ensembl.org) were used for gene expression profiling. Quantification of the mapped RNAseq data was done using the RSEM software suite (v. 1.3.3), obtaining expected and normalized RNAseq read counts (in transcripts per million, TPM). DESeq2 (v. 1.38.3) was used to compare mRNA abundance between samples using the RSEM-calculated expected counts for each gene as input data. For comparing mRNA abundance between TRAP-enriched or FACS-sorted samples versus their corresponding non-enriched or non-sorted input, a generalized linear model (GLM) based on the negative binomial distribution was used, as summarized by the formula [GeneExpression ~ condition], where the condition is either ‘enriched’ (for TRAP-enriched or FACS-sorted mRNAs) and ‘not enriched’ (for TRAP experimental mRNA input or mRNA from dissociated FACS samples, respectively). The Wald test, as implemented by DESeq2, was used to test for statistical significance. For identifying mRNA abundance differences between the two TRAP-enriched samples, i.e., endothelial cell TRAP-enriched mRNAs versus whole animal TRAP-enriched mRNAs, a GLM as summarized by the formula [ GeneExpression ~ experiment + condition + experiment:condition] was used, where ‘experiment’ indicates how mRNAs were isolated (by TRAP-enrichement or input mRNAs), and ‘condition’ indicates the different samples being compared (endothelial cell or whole animal mRNAs). The interaction term ‘experiment:condition’ is, in essence, the ratio of ratios [(TRAP-enriched endothelial cell polysome/input endothelial cell polysome)/(TRAP-enriched whole animal polysome/input whole animal polysome)]. As implemented in DESeq2, the likelihood ratio test was used along with a reduced model that excludes the ‘experiment:condition’ interaction term to test for differences in the TRAP enrichment of mRNAs between endothelial cell and whole animal samples.

For the adult TRAP samples, sequenced FASTQ files were trimmed with cutadapt and aligned with hisat2 using lcdb-wf pipeline (https://github.com/lcdb/lcdb-wf, v1.9rc). We used zebrafish genome assembly version GRCz11 and Ensembl gene annotation version 99. Subsequent downstream analyses were performed with the lcdb-wf pipeline utilizing R and packages DESeq2, clusterProfiler and GeneTonic (for fuzzy clustering). To determine which genes were endothelial enriched within a specific tissue, one-sided Wald t-tests were used to select for genes that were upregulated in the TRAP pulldown (IP) for that tissue compared to its total mRNA lysate (input), later referred to as the IP > Input comparison. To determine which endothelial enriched genes were shared among all tissues and the whole fish control, a multiple testing procedure for multi-dimensional pairwise comparisons was applied [27]. To control the false discovery rate (FDR) across genes at 10%, we extracted raw p-values for each gene and each tissue IP > Input comparison, with missing values being replaced by 1. A Holm correction was applied across comparisons for each gene separately. The FDR was calculated across genes using gene-wise minimal Holm-corrected p-values, and the fraction R of genes with FDR < 0.1 was determined. For each IP > Input comparison we selected genes whose within-gene Holm-corrected p-values were less than 0.1*R (and whose across-genes FDR was less than 0.1). Intersection of these genes across all IP > Input comparisons yielded a set of 350 genes. Out of this list, select genes were picked to be displayed in a heatmap in which their TRAP pulldown log2fold expression for each tissue was ≥ 1.2 compared to their own tissue lysate with a p-adjusted value < 0.05.

To determine which genes were uniquely enriched in the endothelium of specific organs, we pre-screened for genes within each tissue whose endothelial TRAP pulldown (IP) was upregulated compared to its total tissue mRNA lysate (input) and, separately, whose endothelial TRAP pulldown was upregulated compared to the whole fish endothelial TRAP pulldown, using a similar multiple testing procedure for multi-dimensional pairwise comparisons as described above, and taking an intersection of the two comparisons. This yielded a list of 3304 genes combined across all tissues (excluding whole fish). Based on this dataset, we determined genes dominantly expressed in each tissue's TRAP pulldown (IP) sample. The criteria for selection were as follows: (1) a gene was selected in a given tissue during the pre-screening, (2) it passed the omnibus FDR < 0.1 testing overall gene change across IPs (using minimum Holm-corrected p-values of all pairwise IP vs. IP comparisons), (3) statistically verified greater IP expression than in all other tissue IPs (with cross-IP Holm-corrected p-values < 0.1 × R, where R is the fraction of genes passing the omnibus test), (4) the minimum across the tissue IP replicates is above any other IP replicates in all other tissues. Gene lists for each tissue were then further filtered as follows: (1) endothelial gene expression was at least 1.5-fold greater than that of the organ lysate, (2) endothelial gene expression was at least twofold greater than the endothelial gene expression of the next highest tissue, and (3) the difference in endothelial gene expression between the tissue of interest and the next highest expressing tissue was at least twofold greater than their tissue lysate differences. A heatmap was generated using the top 7–8 genes that fit these criteria for each organ. Known information on selected genes was obtained using ZFIN and UniProt databases [28, 29].

### Functional annotation of gene lists determined from RNAseq analyses

Gene Ontology (GO) term enrichment analyses were performed using the PANTHER classification system (v18.0, https://pantherdb.org/). Statistical overrepresentation of biological processes GO terms was done using gene lists that were determined from the RNAseq analyses performed on larval samples. The Fisher’s exact test with Bonferroni correction was used for statistical testing.

### Analysis of gene features

For calculating codon usage, the coding sequences (CDSs) for all annotated transcripts were obtained from Ensembl (release 99, https://ftp.ensembl.org) in FASTA format. Custom Perl scripts were used to retrieve specific transcript sequences and to count codons from these sequences. Only Ensembl CDSs having a length consistent with a genetic code made up of three-nucleotide codons were considered. Additionally, only CDSs with the start codon ATG and stop codons TAA, TAG, and TGA were considered. The selection criteria for a representative annotated transcript for each annotated gene were as follows: (1) The transcript with the longest CDS length was selected as the representative transcript sequence for its corresponding annotated gene, (2) when attempting to select for the longest transcript, if there was a tie in CDS lengths of two or more transcripts for a given annotated gene, the transcript with the longest mRNA length was then selected. Upon selection of the representative CDSs for each annotated genes, the codon usage of ‘more highly translated’ genes and ‘less highly translated’ genes was determined from the Ensembl CDS. Partitioning of genes into one of these two categories was done using the results from the RNAseq analysis between the whole animal TRAP-enriched mRNA versus the corresponding input mRNA. Codon usage was determined by counting the number of times a codon was seen among all CDSs of genes that had higher mRNA abundances in the whole-animal TRAP-enriched samples—the ‘more highly translated’ genes. Likewise, the same codon counting method was used for genes found to have lower mRNA abundances among whole animal TRAP-enriched samples, with these genes being considered ‘less highly translated.’

### Western blot

Protein samples were collected and boiled in Laemmli buffer + 2-mercaptoethanol and run on 12% polyacrylamide gels, transferred onto PVDF membranes, and then blocked in 5% BSA. Blots were probed with either anti-RPL11 (Abcam, Cat. # ab79352, RRID: AB_2042832) or anti-HA (Sigma, H9658, RRID: AB_260092) antibodies. Blots were exposed on film (Amersham Hyperfilm ECL, GE28906836) using Amersham ECL Western Blotting Reagent (GE, RPN2106).

### Fluorescence activated cell sorting

For Fluorescence Activated Cell Sorting (FACS), 24hpf AngioTag embryos were anaesthetized with Tricaine and dechorionated and deyolked. Embryos were then washed in PBS and incubated in 0.25% trypsin at room temperature with trituration until dissociated into a single cell suspension. Following embryo dissociation, trypsin was quenched with Leibovitz L-15 phenol-free media with 10% FBS (Gibco). Cells were passed through a 70 µM strainer and collected by centrifugation at 600xg for 1 min. Cells were resuspended in phenol-free L-15 + 10% FBS, and GFP + cells were collected by FACS sorting on a BD FACSAria™ III machine using BD FACSDiva™ software (Becton Dickinson Biosciences). Sorted cells were collected at 500×*g* for 5 min and RNA was isolated and DNase treated using the ZR-Duet kit (Zymo Research D7003).

### Whole-mount RNA in situ hybridization

DIG-labeled antisense riboprobes for the below genes were generated using DIG Labeling Kit (Roche). In situ hybridization was performed as described [3]. BM purple (Sigma) was used for DIG-labeled probes. Riboprobes were generated for the following genes:

- ENSDARG00000069998 (F′ GGTGCAGATAACTGGGAAGGTGATAG, R′ TCAGTGTGAAGACGTACACC),
- ENSDARG00000076721 (F′ CAGATGAAGTAAAGTCAGTATCTGTGATG, R′ GTAGTCTGGTTGGTGAATGAATAAGC),
- ENSDARG00000098293 (F′ GACCATGTGCTGAGAAATGTGAAGAG, R′ TGTTAGCTCCATTTCCGCAG),
- ENSDARG00000098129 (F′ GCTGTCTGTGGAGCGCTAAGTGTTTGTCT, R′ TAATACGACTCACTATAGGGAGAATGTCACATCCGACCAATCAGAAT),
- ENSDARG00000008414/exoc312a (F′ GAGGCTGAAGGTAGATTTGGACAGATCGAC, R′ TGTGGATCCCACTATTTTTACAGTG),
- ENSDARG00000056643/slc22a7b.1 (F′ ACAACTTTATCGCCGCCATC, R′ AGCCCTCCAGTCATTCACAA),
- ENSDARG00000099980/bpifcl (F′ CAGAAGCAGATGAAGTTCATTAGTTCATTA, R′ CTCCATGTTAGTGACTGCTTGTTGG)

### Hybridization chain reaction (HCR) in situ

HCR probes were designed and purchased from Molecular Instruments or designed using the in-situ-probe-generator_v.0.3.2 [30] and purchased from IDT. The molecular instruments protocol for fixed whole-mount zebrafish embryos and larvae was followed with a few modifications. Whole fish were fixed in 4% paraformaldehyde (PFA) for 2 h at room temperature, with a cavity opened for better PFA penetration, then organs dissected and rinsed in 1 × phosphate-buffered saline. The methanol steps were not performed and instead organs directly underwent proteinase K treatment after the 1 × phosphate-buffered saline rinses which ranged from 30-50µg/ml for 10–15 min depending on the organ. 4 pmol of each probe was utilized for hybridization. Organs were incubated with 1μg/ml of DAPI in 5 × SSCT for one hour before a final 5 × SSCT only wash step.

### Generation of constructs and transgenic lines

The RiboTag and AngioTag constructs were generated using Gateway Technology [31]. Rpl10a was PCR amplified from 24 hpf zebrafish cDNA using forward primer GTG AGA GGG GAG ATA TCA CG and reverse primer CTA AGC GTA ATC TCC AAC ATC GTA TGG GTA GTA GAG GCG CTG TGG TTT TCC CAT G, and TOPO TA cloned into the PCR-II vector. Bridging PCR was then used to add EGFP and viral 2A sequence [32] to generate the “RiboTag” construct pME-egfp-2a-rpl10a-3xHA. Gateway LR reactions were used to combine pDEST-IsceI-Flk7kb, pME-egfp-2a-rpl10a-3xHA, and p3E-polyA. The kdrl:egfp-2a-rpl10a3xHA “AngioTag” construct was digested with IsceI enzyme and microinjected into the blastomere of one-cell stage zebrafish embryos. A stable *Tg(kdrl:egfp-2a-rpl10a3xHA)*^*y530*^ germline transgenic line was established by screening through multiple generations.

The UAS:RiboTag construct was generated using SLiCE technology [32]. The pT1ump-14xUAS-MCS-POUT [34] was digested with EcoRI and XhoI and then forward primer TCC CAT CGC GTC TCA GCC TCA CTT TGA GCT CCT CCA CAC GAA TTC GCC ACC ATG GTG TCA AAA G and reverse primer ACA TGT TCA GTT AAC GGT GGC TGA GAC TTA ATT ACT AGT CTC GAG TTA AGC GTA ATC TGG AAC ATC were used to slice clone the RiboTag cassette downstream from the 14 × UAS sequence, using pME-egfp-2a-rpl10a-3xHA as the template.

The Gene C (ENSDARG00000098293) and Gene B (ENSDARG00000076721) mutants were generated using CRISPR-Cas9 technology, as outlined in Gagnon et al. [35]. For ENSDARG00000098293, the guide primer sequence was TAATACGACTCACTATAGGAATTGGGCGACTTACTGCGTTTTAGAGCTAGAAATAGCAAG, for ENSDARG00000076721, the guide primer sequence was TAATACGACTCACTATAGGTTTGGACCTCATGAGAGTGTTTTAGAGCTAGAA. Genotype was determined using an ABI 3130 (Applied Biosystems) with screening primers TGTAAAACGACGGCCAGTATGGCTGTAGATGAATGAAGACT and GTGTCTTTCTCAGCACATGGTCAGAGG for ENSDARG00000098293 and screening primers TGTAAAACGACGGCCAGTCAGGTGTGTTTGGTGCTGAT and GTGTCTTCACGGGCATTAACTCACCAT for ENSDARG00000076721.

The Gene C mutant described in this manuscript has a 20bp deletion in exon 2.

**Table:** 

^37^TTGGGCGACTTAA—GTGAAGGAGTTAAGCGAAGCTCAGACC^76^	
^37^TTGGGCGACTTACTGCAGGAGTTTAATGATGTTGTGAAGGAGTTAAGCGAAGCTCAGAC^96^	

The Gene B mutant described in this manuscript has a 5bp deletion in exon 2.

**Table:** 

^192^TTATCAGATACTGTGGATGTTTGGACCTCATGAG—GATAGCTGAAATCTATAAGCA^247^	
^192^TTATCAGATACTGTGGATGTTTGGACCTCATGAGAGTCGGATAGCTGAAATCTATAAGCA^252^	

### Microscopic image acquisition and processing

Larvae used for imaging were anesthetized using 168 mg/L Tricaine (1X Tricaine) and mounted in 0.8–1.5% low melting-point agarose dissolved in embryo buffer and mounted on a depression slide. Confocal fluorescence imaging for larvae and adult organs was performed with an LSM 880 (Zeiss) or a Nikon TI2 with CSUW1 spinning disk microscope. Confocal images were processed using either Zen software or Nikon Elements. Schematics and figures were made using Adobe Illustrator 2023 and BioRender software.

### Caudal plexus measurements

The dorsal–ventral width of the caudal plexus was measured under the first ISV posterior to the anal pore, using Fiji [36]. Measurements to the left, underneath, and to the right of the ISV were taken, and then averaged and normalized to the overall dorsal–ventral height of the embryo at the first ISV posterior to the anal pore, which was measured in a similar manner to the caudal plexus. The values for each genotype were then averaged, normalized to the wild-type measurement, and the standard error of the mean was determined. A student’s t-test was run to determine significance.

## Results

### Translating ribosome affinity purification (TRAP) in zebrafish embryos

**Fig. 1:**
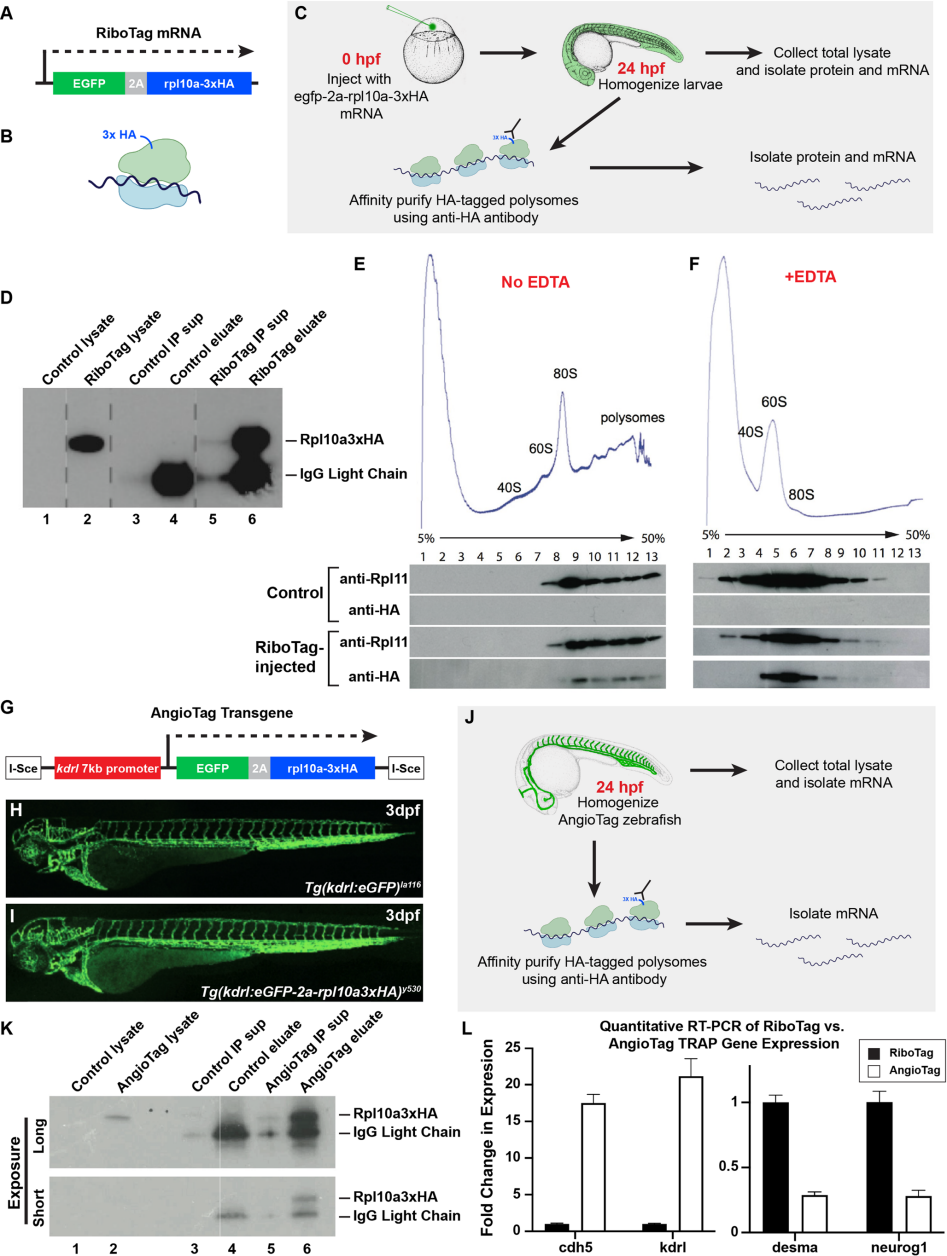
Affinity-tagged ribosomal protein subunits (Ribotags) can be used to purify translated RNAs in vivo. **A** Schematic diagram of the egfp-2a-rpl10a3xHA RiboTag cassette. **B** Schematic diagram of a 3xHA-tagged ribosome translating an mRNA. **C** Schematic diagram illustrating the workflow for TRAP purification of RNAs from RiboTag mRNA-injected zebrafish. **D** Western blot of starting lysates and TRAP supernatants and eluates from either control or RiboTag-injected animals, probed with αHA antibody. IgG light chain is present in the pull-downs. **E** Western blots of fractions collected from sucrose density gradient sedimentation of lysates from control or RiboTag-injected animals, probed with either αRpl11 or αHA. HA-tagged ribosomes sediment together with Rpl11-positive polysomes in the RiboTag-injected samples. **F** Western blots of fractions collected from sucrose gradient sedimentation of EDTA-treated lysates from control or RiboTag-injected animals, probed with either αRpl11 or αHA. Rpl11-positive endogenous ribosomes and HA-tagged ribosomes both sediment in the 60S fraction after EDTA treatment. **G** Schematic diagram of the IsceI(kdrl:egfp-2a-rpl10a3xHA) transgene. **H** Composite confocal micrograph of a 3dpf *Tg(kdrl:egfp)*^*la116*^ transgenic animal, for comparison purposes. **I** Composite confocal micrograph of a 3dpf *Tg(kdrl:egfp-2a-rpl10a3xHA)*^*y530*^ (“AngioTag”) transgenic animal. **J** Schematic diagram illustrating the workflow for TRAP purification of RNAs from AngioTag zebrafish. **K** Western blot of starting lysates and TRAP supernatants and eluates from either control or AngioTag animals, probed with αHA antibody, showing longer (top) and shorter (bottom) exposures of the same blot. IgG light chain is present in the pull-downs. **L** Quantitative RT-PCR measurement of the relative expression of endothelial genes *cdh5* and *kdrl*, and non-endothelial genes *desma* and *neurog1* in cDNA samples prepared from TRAP purified RNA from either RiboTag control (black columns) or AngioTag (white columns) animals, showing enrichment of vascular specific genes and depletion of non-vascular specific genes in the AngioTag TRAP samples

To determine whether TRAP could be used to isolate polysome-bound mRNAs from developing zebrafish, we designed an egfp-2a-rpl10a3xHA RiboTag cassette. This cassette includes the 60S ribosomal protein L10a (rpl10a) fused to triplicate hemagglutinin (HA) epitope sequences (rpl10a3xHA). To visualize transgene expression, the cassette also includes EGFP linked to rpl10a3xHA via a viral 2A cleavage site [32], permitting bicistronic expression of separate EGFP and rpl10a3xHA polypeptides (Fig. 1A). Assembly of Rpl10a3xHA protein into functional ribosomes marks translating mRNAs in cells expressing this transgene (Fig. 1B). To test whether the transgene could be employed to isolate tagged polysomes using TRAP, we injected RiboTag mRNA into single-cell embryos, raised the embryos to 24 hpf, homogenized them, and affinity-purified HA-tagged polysomes using αHA antibody followed by direct immunoprecipitation with Dynabeads® (Fig. 1C). Western blotting of starting lysates, post-immunoprecipitation supernatants, and eluates from αHA pull-downs (Fig. 1D) showed that Rpl10a3xHA protein was readily detected in RiboTag-injected (lane 2) but not in control uninjected (lane 1) embryos, and that Rpl10a3xHA protein was efficiently pulled down using the αHA antibody (lane 6) and depleted from the supernatant (lane 5).

To ensure that Rpl10a3xHA protein is properly incorporated into the 60 S ribosomal subunit, the 80 S ribosome, and polysomes, we prepared lysates from RiboTag mRNA-injected and control-uninjected embryos and performed velocity sedimentation in sucrose density gradients, followed by Western blotting of collected fractions (Fig. 1E-F). We probed the blots using an antibody-recognizing endogenous Rpl11, a component of the 60 S subunit, and found that Rpl11 sediments similarly within the 60 S, 80 S, and polysome fractions in either uninjected controls or RiboTag-injected animals (Fig. 1E). When we probed the blots using an αHA antibody, we observed similar sedimentation patterns for Rpl10a3xHA protein in the RiboTag-injected animals, leading us to conclude that the epitope tag does not interfere with assembly of the 60 S subunit, 80 S ribosome, or polysomes (Fig. 1E). As expected, disassembly of polysomes using EDTA treatment causes both Rpl11 and Rpl10a3xHA to elute only in the 60 S fraction; they are not seen in the 80 S or polysome fractions (Fig. 1F). These results show that Rpl10a3xHA protein assembles into ribosomes in vivo and can be used for isolation of tagged polysomes from zebrafish.

### Isolating endothelial-specific RNA using AngioTag zebrafish

We next set out to create transgenic zebrafish expressing the RiboTag construct specifically within endothelial cells. We utilized the previously published endothelial specific 7 kb *kdrl* promoter [22] to design a IsceI(kdrl:egfp-2a-rpl10a3xHA) AngioTag transgene expressing the RiboTag cassette in an endothelial cell type-specific manner (Fig. 1G). We obtained a strongly expressing *Tg(kdrl:egfp-2a-rpl10a3xHA)*^*y530*^ germline transgenic line that shows a vascular-restricted EGFP expression closely matching the expression pattern of a previously generated *Tg(kdrl:egfp)*^*la116*^ [22] transgenic line driving EGFP alone (Fig. 1H–I). To test whether this AngioTag transgenic line could be used for TRAP enrichment of endothelial mRNAs, we prepared homogenates from 24 hpf AngioTag or control non-transgenic animals and subjected the lysates to the TRAP procedure (Fig. 1J). Western blotting of starting lysates (lanes 1 and 2), TRAP supernatants (lanes 3 and 5), and TRAP eluates (purified tagged polysomes, lanes 4 and 6) from non-transgenic (lanes 1,3,4) or AngioTag transgenic (lanes 2,5,6) animals (Fig. 1K) showed that Rpl10a3xHA protein was readily detected in AngioTag (lane 2) but not in control (lane 1) non-transgenic total lysates, and that Rpl10a3xHA was pulled down from AngioTag lysates using αHA antibody (lane 6). To assess whether the AngioTag TRAP procedure resulted in selective enrichment of endothelial-specific mRNAs, we performed quantitative RT-PCR for endothelial and non-endothelial genes using RNA collected from either 24hpf AngioTag transgenic animals or 24 hpf RiboTag mRNA injected animals. RNA isolated from AngioTag animals was highly enriched for endothelial-specific genes *(cdh5, kdrl)* and depleted of genes expressed specifically in non-vascular tissues *(desma, neurog1)* compared to the RiboTag mRNA-injected control RNA samples (Fig. 1L).

### Expression profiling using TRAP-RNAseq in zebrafish

**Fig. 2:**
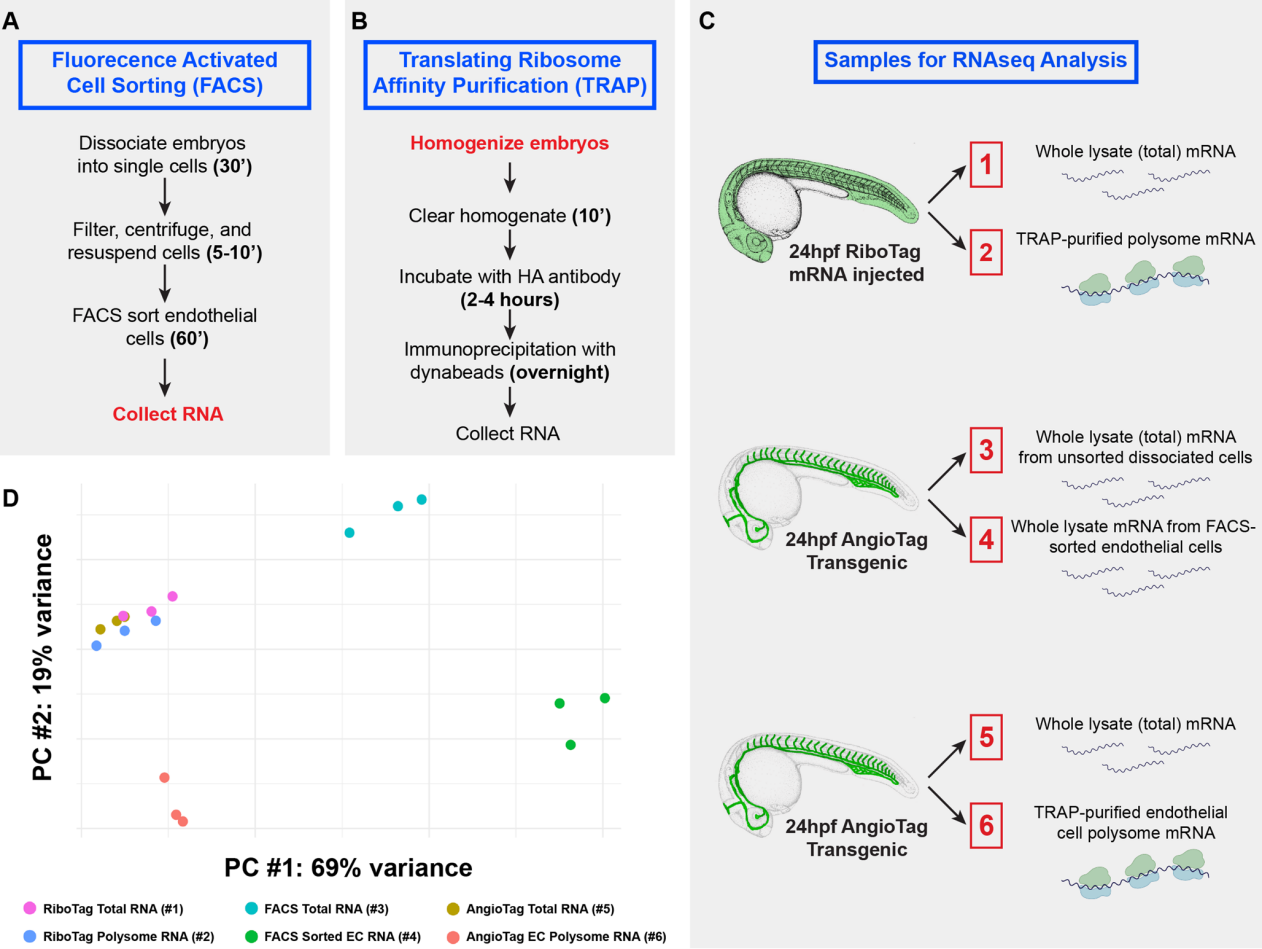
Comparative workflow for preparation of endothelial-specific RNAs using fluorescence activated cell sorting (FACS) versus translating ribosome affinity purification (TRAP). **A** Workflow for FACS of EGFP-positive endothelial cells and RNA preparation from 24hpf *Tg(kdrl:egfp-2a-rpl10a3xHA)*^*y530*^ AngioTag transgenic animals (sample #4 in panel C). RNA is collected from the cells after more than 1½ hours of embryonic dissociation and cell sorting. **B** Workflow for TRAP of translated mRNAs from 24hpf egfp-2a-rpl10a3xHA RiboTag mRNA-injected animals (sample #2 in panel C) or *Tg(kdrl:egfp-2a-rpl10a3xHA)*^*y530*^ (AngioTag) transgenic animals (sample #6 in panel C). Lysates are prepared from intact animals, and the RNA is stabilized immediately at the beginning of the procedure. **C** Samples collected in triplicate for RNAseq analysis. 24hpf RiboTag mRNA injected (samples 1 and 2), dissociated AngioTag transgenic (samples 3, 4), or AngioTag transgenic animals (samples, 5, 6). Part of each sample was used for whole lysate total RNA collection (samples 1, 3, and 5). The remainder of each sample was used for either TRAP purification of total (sample 2) or endothelial (sample 6) polysome mRNA, or for FACS sorting of EGFP-positive endothelial cells followed by RNA isolation (sample 4). **D** Principal component analysis of RNAseq data obtained from the six sample types noted in panel C, each run in triplicate (total of 18 samples). The greatest variance is seen between those samples in which cells were dissociated (samples 3 and 4) and those that were not (samples 1, 2, 5, 6)

Having shown that our AngioTag transgenic line could be used to selectively enrich for endothelial gene expression, we wanted to compare AngioTag TRAP-RNAseq endothelial expression profiling to FACS endothelial expression profiling using previously published methods in zebrafish [37–39] (Fig. 2A–B). As noted above, FACS protocols require the dissociation of an embryo into a single cell suspension, disrupting cell–cell, cell–matrix, and other external interactions for a prolonged period prior to cell lysis and RNA collection (Fig. 2A). In contrast, the TRAP procedure involves immediate homogenization, cell lysis, and stabilization of translating RNAs (Fig. 2B), providing an instant ‘snapshot’ of translated gene expression at the time of embryo homogenization, one that is potentially more representative of an ‘in vivo’ profile.

We performed RNAseq in triplicate on a total of six different sample types (Fig. 2C). RNA was prepared from TRAP-purified polysomes from 24hpf RiboTag mRNA injected animals (Sample 2, ‘whole TRAP translatome’), from FACS-sorted endothelial cells from 24hpf AngioTag transgenic animals (Sample 4, ‘endothelial FACS transcriptome’), and from TRAP-purified polysomes from 24hpf AngioTag transgenic animals (Sample 6, ‘endothelial TRAP translatome’). For each of these samples, we also collected control RNA from either the starting lysates used for TRAP (Samples 1 and 5) or from unsorted cells (Sample 3). Three experimental replicates were performed for each of these sample types from separately collected samples, for a total of 18 samples used for RNAseq analysis. A minimum of 45 million reads were obtained for each sample, with an average of 84% of reads mapping uniquely (Supplemental Table 1). All sequence data was deposited with GEO (accession # GSE292080). Principal component analysis (Fig. 2D) showed that the RiboTag-TRAP (Samples 1 and 2) and AngioTag-TRAP (Samples 5 and 6) starting lysates and TRAP samples clustered fairly close together, while the FACS unsorted (Sample 3) and sorted (Sample 4) cell samples showed considerable divergence both from the other samples and from one another. This suggests that cell dissociation and cell sorting can cause changes in gene expression that are distinct from endogenous expression for these cells.

**Fig. 3:**
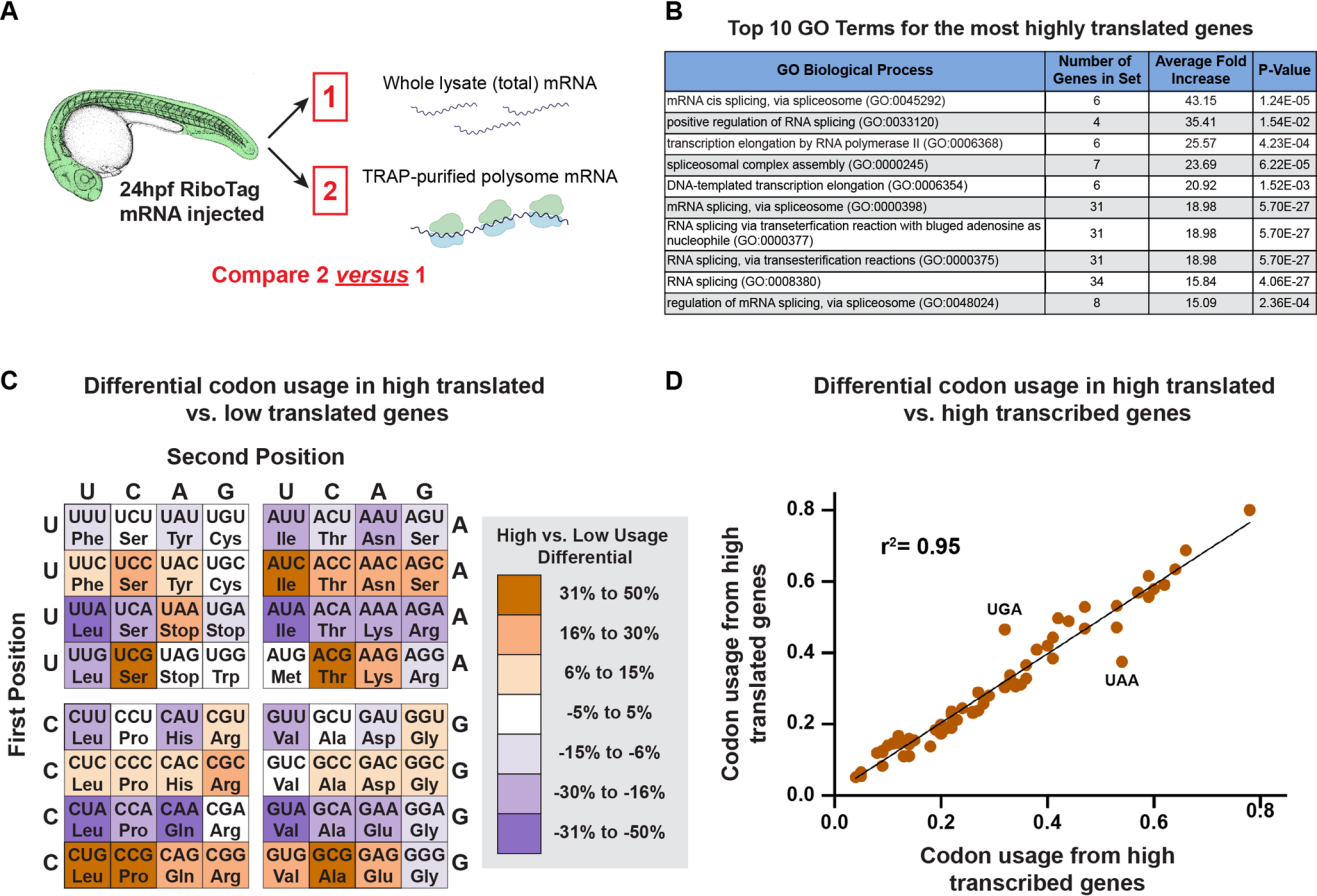
Comparison of the whole-animal transcriptome to its translatome. **A** Samples collected for RNAseq analysis of whole animal translatome (sample 2) vs. whole animal transcriptome (sample 1) from 24 hpf RiboTag mRNA injected animals. **B** Top 10 GO terms for the highest-translated genes based on average fold increase. **C** Percent change of codon usage between the most highly translated genes (log2(fold) > 0.35, BH-adjusted *p* < 0.05 comparing samples 2 vs. 1) and the least highly translated genes (log2(fold) < − 0.35, BH-adjusted *p* < 0.05 comparing samples 2 vs. 1). The color-coding scheme shows the percent change in the relative usage of each codon between the two sets of genes with the usage of each codon calculated as a percentage of the total number of codons coding for a particular amino acid. **D** Scatter plot comparing our findings of codon usage to those discussed in Horstick et al. [40]. The plot shows strong correlation between codon usage from high translated genes in our data set and high transcribed genes from Horstick et al. (r^2^ = 0.95)

Comparing the whole-embryo lysate transcriptome (Sample 1) and matched whole-embryo TRAP translatome (Sample 2) from animals ubiquitously expressing RiboTag (Fig. 3A, Supplementary Material File 2) showed that 1430 genes were significantly differentially represented in the two RNAseq datasets (Benjamini-Hochberg -adjusted p < 0.05), with 807 genes showing increased representation (log2(fold) > 0.35) and 623 genes showing reduced representation (log2(fold) < -0.35) in the whole-embryo TRAP translatome (Sample 2) vs. the transcriptome (Sample 1) datasets. Eight of the ten PANTHER overrepresentation test [40] GO terms with the largest fold increase in the whole-animal translatome (Sample 2) vs. the transcriptome (Sample 1) were related to splicing, suggesting mRNAs for these genes may be more highly translated (Fig. 3B). To determine whether there are sequence features associated with increased or decreased translation, we examined relative codon usage associated with genes over- or under-represented in sample 2 vs. sample 1. By calculating the change in codon usage between the two groups, we identified strong preferences for and against particular codons in high- versus low-translated genes (Fig. 3C). Interestingly, our findings showing codon usage bias for highly translated genes closely correlates (r^2^ = 0.95) with previously published data examining codon usage bias in highly transcribed genes in the zebrafish genome [41] (Fig. 3D), with only slight differences seen in stop codon usage. These findings suggest that there is preferential codon usage for genes being highly translated which in turn can affect protein expression efficiency.

### AngioTag profiling endothelial genes

**Fig. 4:**
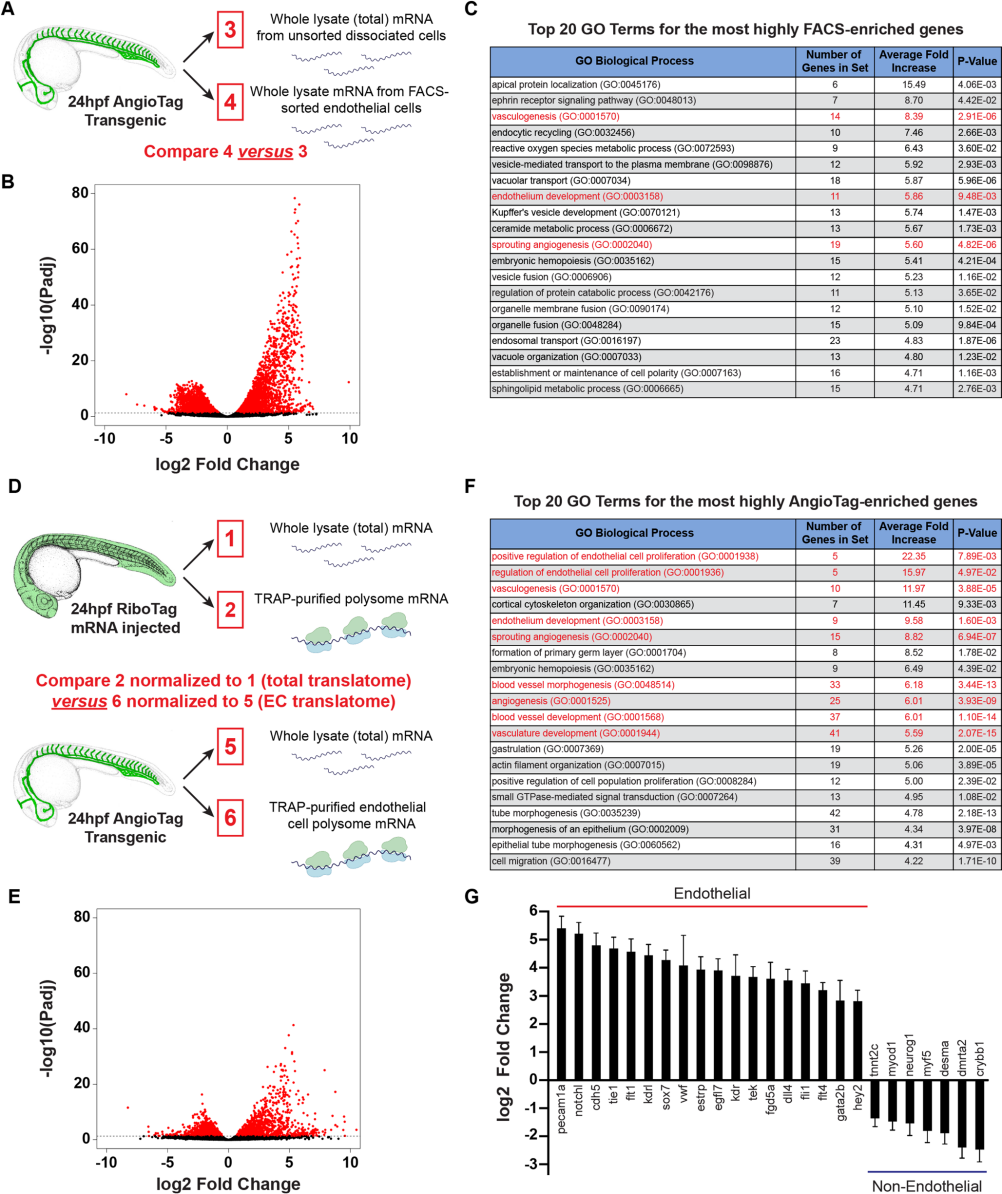
Translating Ribosome Affinity Purification shows greater endothelial gene enrichment compared to Fluorescence Activated Cell Sorting. **A** Samples collected from 24 hpf AngioTag transgenic animals dissociated into a cell suspension, for RNAseq analysis of mRNA from either FACS sorted endothelial cells (sample 4) or unsorted input cells (sample 3). **B** Volcano plot of endothelial enrichment using the sample comparison shown in panel A. **C** Top twenty GO terms for genes most highly enriched in the FACS sorted endothelial cell samples vs. whole animal dissociated unsorted cells. Three out of the top twenty GO terms represent endothelial process-related terms (in red text). **D** Samples collected for RNAseq analysis of TRAP purified endothelial cell polysome mRNA from 24 hpf AngioTag transgenic animals (sample 6) normalized to its input total mRNA (sample 5) compared to TRAP purified total embryonic polysome mRNA from 24 hpf RiboTag mRNA injected animals (sample 2) normalized to its input total mRNA (sample 1). **E** Volcano plot of endothelial enrichment using the sample comparison shown in panel D. **F** Top twenty GO terms for genes most highly enriched in the TRAP purified endothelial polysome mRNA samples. Nine out of the top twenty GO terms represent endothelial process-related terms (in red text) compared to only three seen with FACS sorted endothelial cells in panel C. **G** Log2 fold enrichment of endothelial specific genes and depletion of non-endothelial specific genes in AngioTag TRAP-RNAseq sample 6 (normalized to 5) as compared to RiboTag TRAP-RNAseq sample 2 (normalized to 1)

Having shown that TRAP-RNAseq can be used effectively to profile translating mRNAs in vivo in the zebrafish, we next sought to examine whether our AngioTag transgenic line could be used for specific profiling of endothelium in vivo*.* As noted above, FACS has been used previously to isolate endothelial cells from developing zebrafish for gene expression profiling [37–39], so for purposes of comparison, we began by carrying out RNAseq analyses on mRNA from endothelial cells isolated from 24 hpf AngioTag animals by FACS (Fig. 4A–C). RNAseq experiments were carried out on mRNA isolated from total dissociated cells from 24 hpf AngioTag embryos (Sample 3) and on mRNA from EGFP-positive endothelial cells FACS-sorted from the same dissociated cell population (Sample 4; Fig. 4A). Comparative analysis of genes represented in the two RNAseq datasets showed that 3363 genes were significantly enriched in the FACS-sorted endothelial cells (log2(fold) > 0.35, BH-adjusted p < 0.05), while 3106 genes showed significant depletion in the FACS-sorted endothelial cell population compared to the total dissociated cell population (log2(fold) < -0.35, BH-adjusted p < 0.05; Fig. 4B, Supplementary Material File 3). Three out of the top 20 PANTHER overrepresentation test GO terms showing the highest fold enrichment in FACS-sorted endothelial cells were endothelial/vascular-related terms, while the other 17 most highly enriched GO terms were non-endothelial (Fig. 4C).

Next, we examined the results of profiling the endothelium in the AngioTag line using TRAP-RNAseq (Fig. 4D–G). We compared RNAseq datasets from the whole animal translatome (Sample 2) and endothelial translatome (Sample 6) RNAseq datasets after normalization of each of these samples to their whole-animal starting lysate transcriptome (samples 1 and 5, respectively; Fig. 4D, Supplementary Material File 4). 1365 genes were enriched in the endothelial translatome (log2(fold) > 0.35, BH-adjusted *p* < 0.05), while 1141 genes show some depletion when compared to the whole animal translatome (log2(fold) < − 0.35, BH-adjusted *p* < 0.05; Fig. 4E). Of the top twenty GO terms enriched in the endothelial TRAP-RNAseq dataset, nine were endothelial/vascular-related terms (Fig. 4F), a substantially higher number than found in the FACS-sorted endothelial cell data (Fig. 4C). Although three of these nine GO terms were also present in the top twenty terms for the FACS-sorted endothelial cells, they showed greater fold-enrichment in the TRAP-RNAseq dataset (compare Fig. 4C to F). In addition, direct comparisons between the transcriptomes of whole-animal dissociated cells and immediately lysed whole animals (Sample 3 versus Sample 1) or between FACS-sorted endothelial cell mRNA and TRAP-purified endothelial mRNA (Sample 4 versus Sample 6) showed a strong increase in known stress induced genes in the cell-dissociated samples [41], including Fos family members (fos), Heat Shock 70 proteins (hsp70), growth arrest and DNA-damage-inducible expression (gadd45), as well as the early growth response gene (egr1) and activating transcription factor (atf3) (Supplemental Fig. 1, Supplementary Material Files 5 and 6), suggesting that cell dissociation and cell sorting protocols induce stress-related transcriptional changes. Together, these findings suggest that TRAP-RNAseq provides a better readout of in vivo gene expression for cells of interest than FACs-RNAseq. As expected, several known endothelial-specific genes are highly enriched in the AngioTag TRAP-RNAseq dataset including *cdh5* and *kdrl*, while genes that are not expressed in endothelium such as *desma* and *neurog1* are depleted, confirming results obtained by RT-PCR and now confirmed using RNAseq approaches (compare Figs. 1L to 4G).

**Fig. 5:**
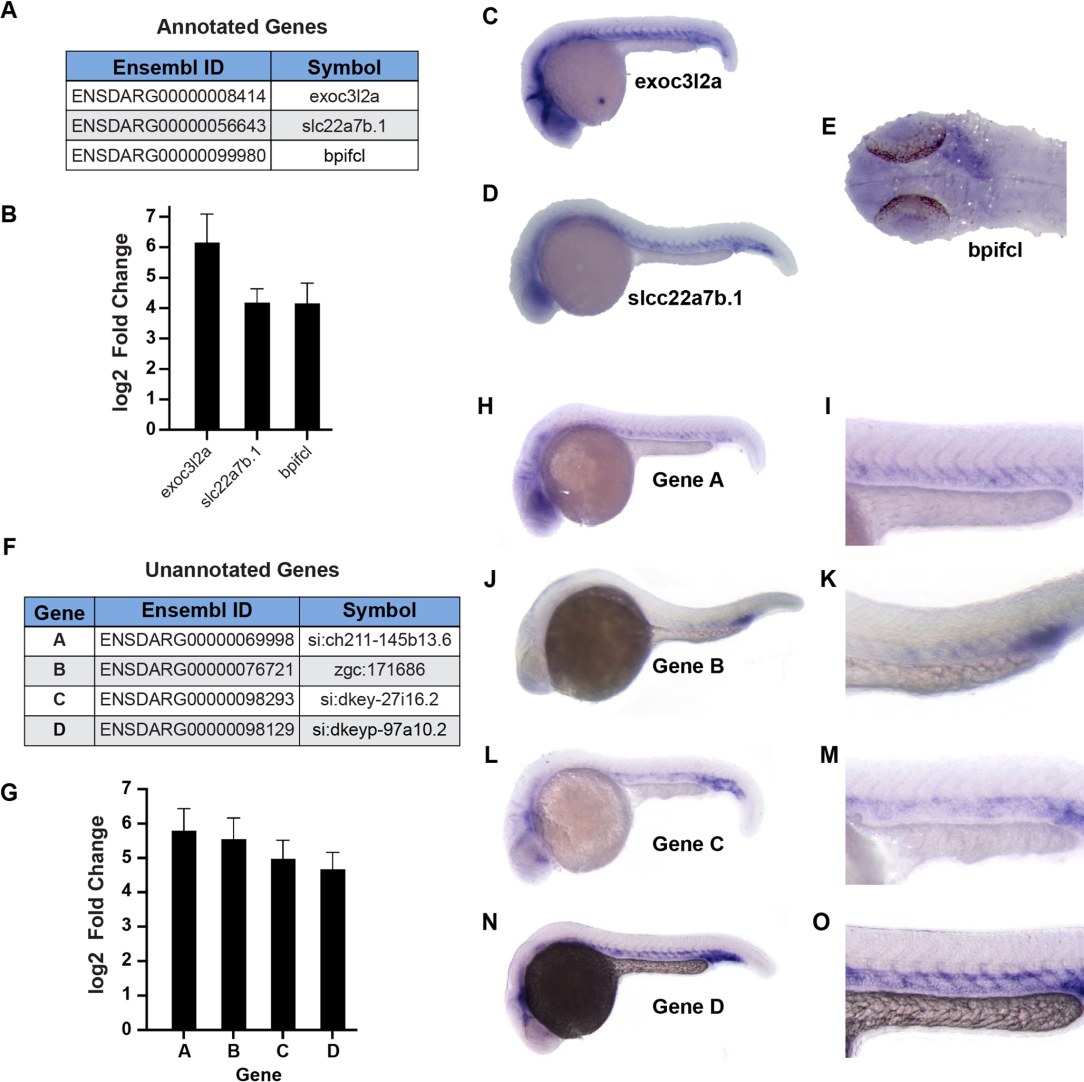
TRAP-RNAseq reveals novel endothelial genes expressed in early vascular development. **A** Three annotated genes enriched in the AngioTag TRAP-RNAseq dataset (sample 6) without previously recognized endothelial expression. **B** Log2 fold enrichment of the three annotated genes noted in panel A in AngioTag TRAP-RNAseq sample 6 (normalized to 5) compared to RiboTag TRAP-RNAseq sample 2 (normalized to 1). **C–E** Whole mount in situ hybridization of 24 hpf wild type zebrafish probed for *exoc312a* (C), *slcc22a7b.1* (D), and *bpifcl* (E). Images shown are either lateral views of the whole animal (panels C-D), or a dorsal view of the head with staining of the heart noted (E). **F** Four unannotated genes enriched in the AngioTag TRAP-RNAseq data set (sample 6). **G** Log2 fold enrichment of the four unannotated genes noted in panel F in AngioTag TRAP-RNAseq sample 6 (normalized to 5) compared to RiboTag TRAP-RNAseq sample 2 (normalized to 1). **H–O** Whole mount in situ hybridization of 24 hpf wild type zebrafish probed for unannotated gene A (panels H-I), gene B (panels J-K), gene C (panels L-M), and gene D (panels N–O). Images shown are lateral views of the whole animal (panels H, J, L, N), with higher magnification lateral views of the trunk (panels I, K, M, O)

**Fig. 6:**
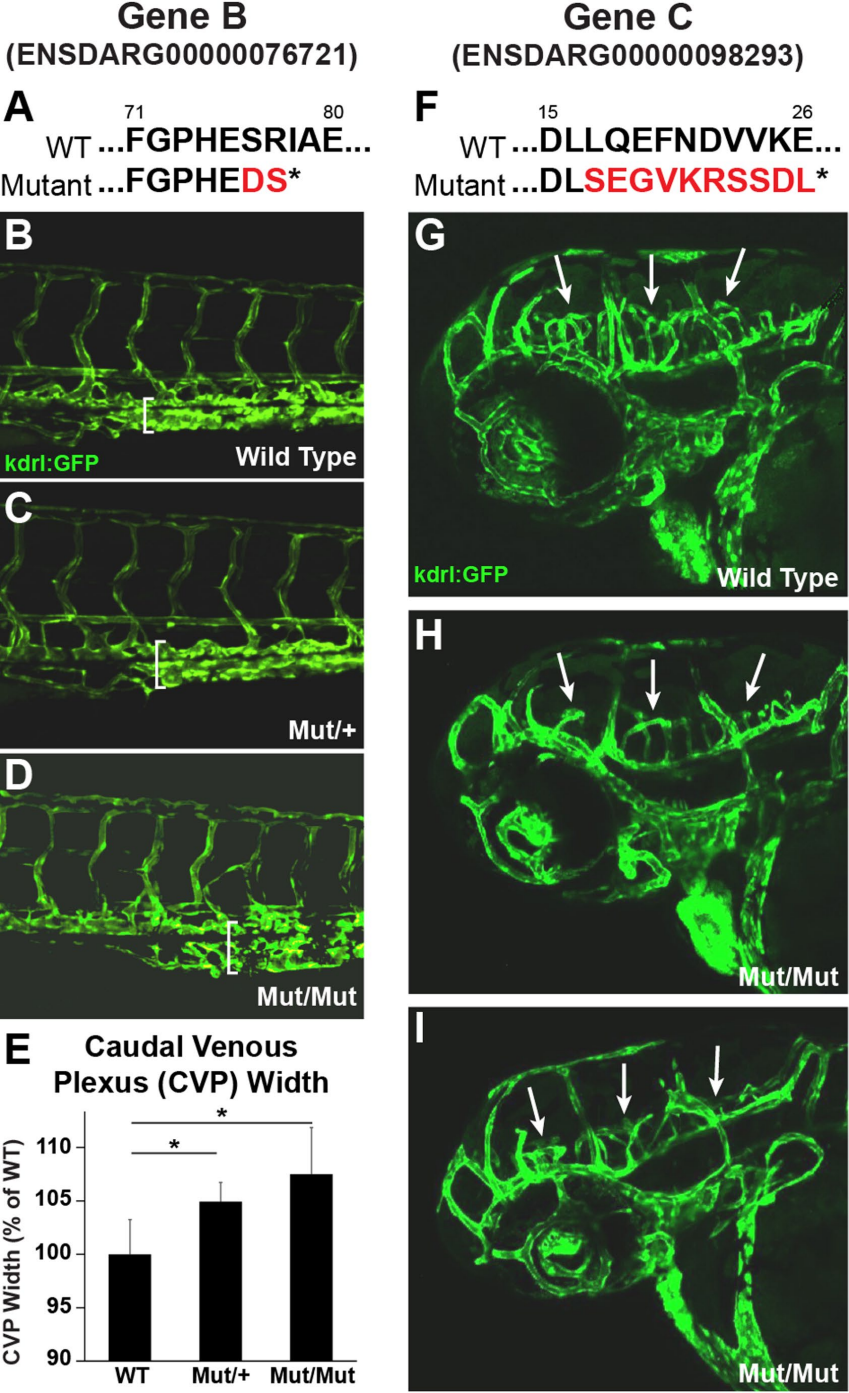
Mutants of unannotated endothelial genes have vascular phenotypes**. A** In *76721*^*y587*^ mutants a 5 bp deletion in ENSDARG00000076721 introduces an early stop codon, resulting in a protein truncation from 316 amino acids to 78 amino acids. **B–D** Confocal microscopy of 3dpf embryos from a *76721*^*y587/*+^ heterozygous in-cross showing a dilated caudal plexus in heterozygous (**C**) and homozygous mutant (**D**) embryos as compared to wild type siblings (**B**). **E** Graph comparing the caudal plexus height of wild type, *76721*^*y587/*+^ heterozygous, and *76721*^*y587/y587*^ homozygous mutant siblings. Values shown are the percent change compared to wild type (N = 41 wild types, 58 heterozygotes, 23 homozygous mutants). **F** In *98293*^*y588*^ mutants a 20 bp deletion in ENSDARG00000098293 introduces an early stop codon, resulting in a protein truncation from 104 amino acids to 27 amino acids. **G–I** Confocal microscopy of 3dpf wild type (G) and *98293*^*y588/y588*^ homozygous mutant embryos (**H**–**I**) reveals decreased cranial vasculature in mutant embryos compared to wild type (white arrows). * student’s t-test *p*-value ≤ 0.05

The vast majority of genes highly enriched by AngioTag TRAP-RNAseq are annotated, and many of these genes are already known to be expressed in the endothelium. However, our enriched gene set included genes not previously reported to be expressed in endothelium (Fig. 5A–B). In situ hybridization for three of these genes– *exoc3l2a, slc22a7b.1,* and *bpifc*–*,* confirmed their endothelial-specific expression pattern (Fig. 5C-E), suggesting that these genes may be involved in vascular development. In addition to annotated genes, our enriched gene set also includes unannotated genes lacking a previously reported link to endothelium, including four highly enriched genes in our dataset (Fig. 5F), each of which shows at least 20-fold endothelial enrichment (log2(fold) > 4) (Fig. 5G). Whole mount in situ hybridization confirms that all four of these genes have highly endothelial-specific expression patterns (Fig. 5H–O), with expression of one of the genes largely restricted to the endothelium of the caudal vascular plexus (Fig. 5J–K). To explore the functional significance of a few of the unannotated genes, we used CRISPR/Cas9 technology to generate targeted mutations in Gene B (ENSDARG00000076721) and in Gene C (ENSDARG00000098293) (Fig. 6). We isolated a 5 base pair deletion mutant in Gene B (76721^y587^), generating a polypeptide truncated after 77 amino acids (Fig. 6A), and a 20 base pair deletion mutant in Gene C (98293^y588^), which coded for a polypeptide truncated after 25 amino acids (Fig. 6F). Homozygous 76721^y587^ mutants display a mild enlargement of the caudal vascular plexus (Fig. 6B–E), while homozygous 98293^y588^ mutants show reduced growth of mid- and hind-brain cranial central arteries (Fig. 6G–I). These results suggest that at least two of the four novel, unannotated genes from this study have roles in early vascular development.

### AngioTag profiling of adult zebrafish organs

**Fig. 7:**
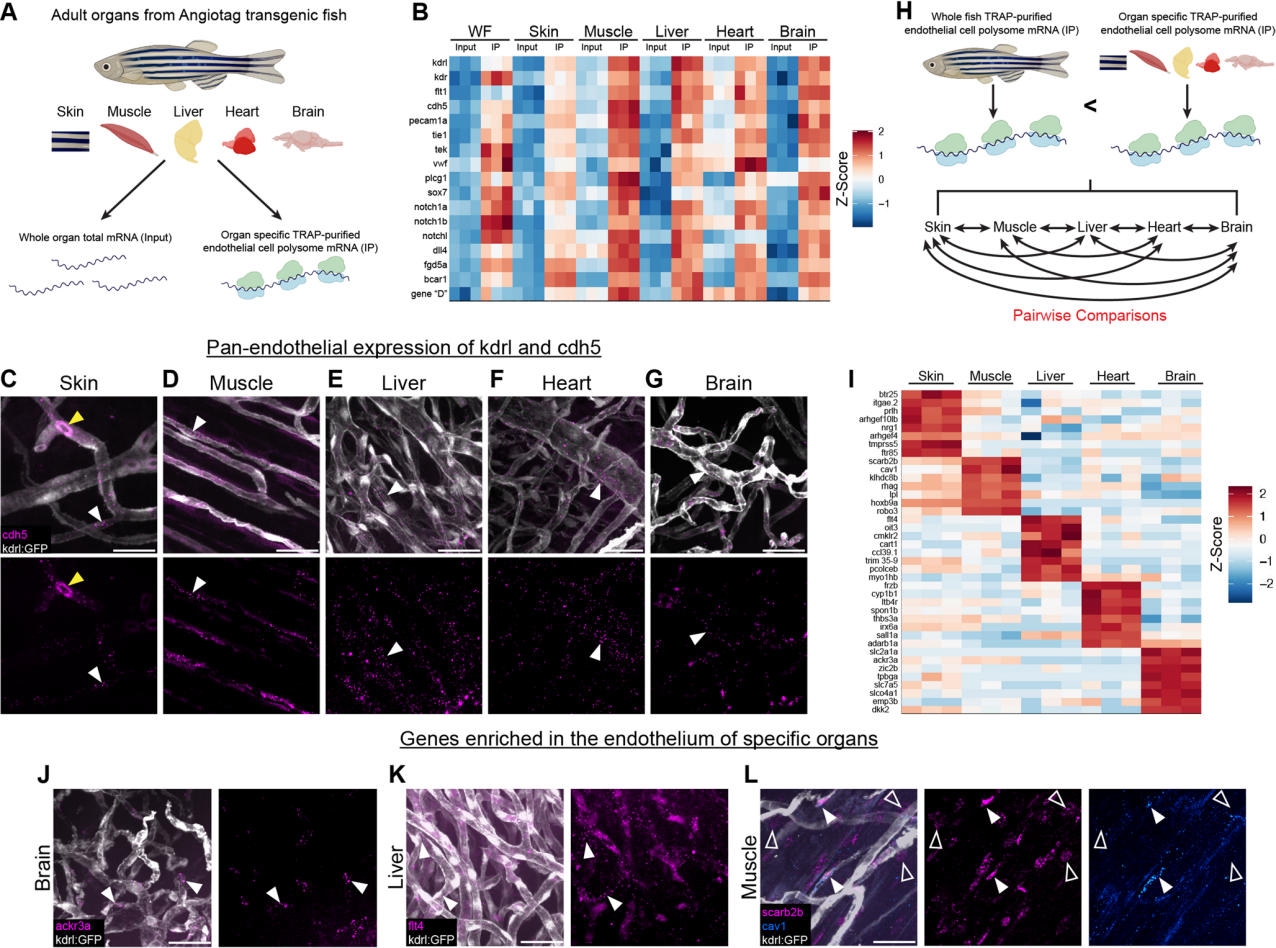
Endothelial profiling using TRAP-RNAseq reveals common vasculature signatures and unique gene expression profiles across vascular beds of different organs**. A** Samples collected for RNAseq analysis of TRAP purified endothelial cell polysome mRNA from five different organs, skin, muscle, liver, heart, brain, and whole fish of AngioTag transgenic animals normalized to each organ’s input total mRNA. **B** Heatmap displaying common endothelial genes enriched in the vasculature of the whole fish (WF) and across vascular beds of each organ compared to their input controls colored by their gene-wise z-scores of the log-transformed normalized counts. TRAP pulldown (IP) and total organ mRNA (input) for each replicate for each organ are shown. **C–G** Confocal images of adult *Tg(kdrl:egfp)*^*la116*^ transgenic fish with hybridization chain reaction (HCR) in situ of *cdh5* showing expression of *kdrl* (white) and *cdh5* (magenta) in the vasculature of the skin (**C**), muscle (**D**), liver (**E**), heart (**F**), and brain (**G**). Merged and *cdh5* only channels are shown. White arrowheads indicate *cdh5* transcript expression while yellow arrowheads show blood cell autofluorescence. **H** Schematic depicting screening steps used to determine genes unique to the vascular beds of each organ. To be included genes first had to be enriched in the vasculature of an organ compared to its total tissue and vascular expression in that organ had to be greater than vascular expression in the whole fish. Gene sets then underwent pairwise comparisons between each organ to determine if they were shared or unique to a particular organ. **I** Heatmap displaying unique endothelial genes enriched in the vascular beds of each organ colored by their gene-wise z-scores of the log-transformed normalized counts. TRAP pulldowns (IP) for each replicate for each organ are shown. **J–L** Confocal images of adult *Tg(kdrl:egfp)*^*la116*^ transgenic fish (white) with hybridization chain reaction (HCR) in situ of *ackr3a* (magenta) in the brain (J), *flt4* (magenta) in the liver (K), and *scarb2b* (magenta) and *cav1* (blue) in the muscle (L). White solid arrowheads indicate probe expression in the vessel, and white open arrowheads indicate probe expression in the muscle fiber. Scale bars are 25μm

In addition to profiling genes involved in vascular development we wanted to know if our AngioTag transgenic fish could be used to reveal tissue-specific differences in the endothelial translatomes of different adult organs (Fig. 7). To test this, we carried out TRAP-RNAseq on skin, muscle, liver, heart, and brain dissected from adult AngioTag transgenic fish, as well as on a whole-AngioTag fish control. Each tissue sample was run in triplicate and each replicate contained organs from four adult fish. Angiogenesis and blood vessel morphogenesis were among the top ten GO terms for each organ’s TRAP pulldown as compared to their whole organ total input (Supplemental Tables 2–6). To uncover endothelial genes common to all vascular beds, we compared the endothelial translatome of each organ (IP) to its whole organ total (input) mRNA, then looked for genes that showed significant enrichment across all of the organ datasets (log2(fold) ≥ 1.2, padj ≤ 0.05) (Fig. 7A, Supplementary Material File 7). As expected, we saw enrichment of well-known endothelial markers such as *kdrl*, *cdh5*, and several Notch pathway members (Fig. 7B). We verified robust expression of *kdrl* and *cdh5* in all five organs using the *Tg(kdrl:egfp)*^*la116*^ transgenic line and whole mount hybridization chain reaction (HCR) *in situs* for *cdh5* (Fig. 7C–G). Our AngioTag data also revealed strong cross-organ endothelial enrichment of some less well-known vascular genes, including *fgd5a*, *bcar1,* and our unannotated Gene D (Fig. 7B).

We carried out an additional two-step comparison to uncover genes uniquely enriched in the endothelium of specific organs (Fig. 7H; see methods for full details). We began by first selecting all genes that showed significantly greater enrichment in the endothelial translatomes of specific organs than in the whole fish endothelial translatome (Fig. 7H, top). The selected genes were then subjected to further pairwise comparisons between each of the organs to find those more highly enriched in one particular organ compared to all of the others (Fig. 7H, bottom). For a gene to be considered unique to the endothelium of a particular organ it had to meet each of the following criteria: 1) endothelial gene expression is at least 1.5 log2(fold) greater than in the starting lysate of that organ, 2) endothelial enrichment in the most-enriched organ is at least 2 log2(fold) greater than in the next-most-enriched organ, and 3) the difference in endothelial gene expression between most-enriched organ and next-most-enriched organ is at least 2 log2(fold) greater than their tissue lysate differences. Applying these very stringent criteria resulted in a small list of genes uniquely enriched in the vasculature of each organ with the top 7–8 genes that fit these criteria for each organ being displayed (Fig. 7I). As expected, the well-known blood–brain barrier gene *slc2a1a*, also known as *glut1*, was highly expressed in the brain vasculature only. The *atypical chemokine receptor 3a* (*ackr3a*) gene was also specifically enriched in the brain vasculature, as validated using HCR *in situs* (Fig. 7I–J). Interestingly, the top GO terms for liver endothelium were lymph angiogenesis, lymph vessel morphogenesis, and lymph vessel development, despite the fact that we were profiling blood vessels, not lymphatic vessels (Supplemental Table 4). In keeping with this finding, we found that the lympho-venous marker *flt4* was selectively enriched in the vasculature of the liver compared to other organs (Fig. 7I). HCR *in situs* for *flt4* in liver tissue confirmed its co-localization with *Tg(kdrl:egfp)*^*la116*^ transgene-positive blood vessels (Fig. 7K). The expression of lymphatic endothelial genes in the liver blood vasculature may be indicative of similarities in vessel function, since lymphatic capillaries are highly permeable and liver vessels are thought to be one of the most permeable types of blood vessels [43, 44]. As has been previously reported using single-cell RNAseq methods in mice [45], we also found organ-specific genes whose expression was shared between both the endothelium and surrounding cells. In muscle, the *scavenger receptor class B, member 2b* (*scarb2b*) gene, predicted to encode a lysosomal membrane protein [29], was expressed in both muscle endothelial cells and in muscle fibers (Fig. 7L), despite expression of other genes such as *caveolin 1* (*cav1*) being restricted to only muscle vessels (Fig. 7L).

Together, our results suggest that TRAP-RNAseq using our AngioTag transgenic line is highly effective for in vivo profiling of endothelial gene expression during development as well as in the vascular beds of adult organs.

### A RiboTag reporter line for profiling any cell or tissue of interest

**Fig. 8:**
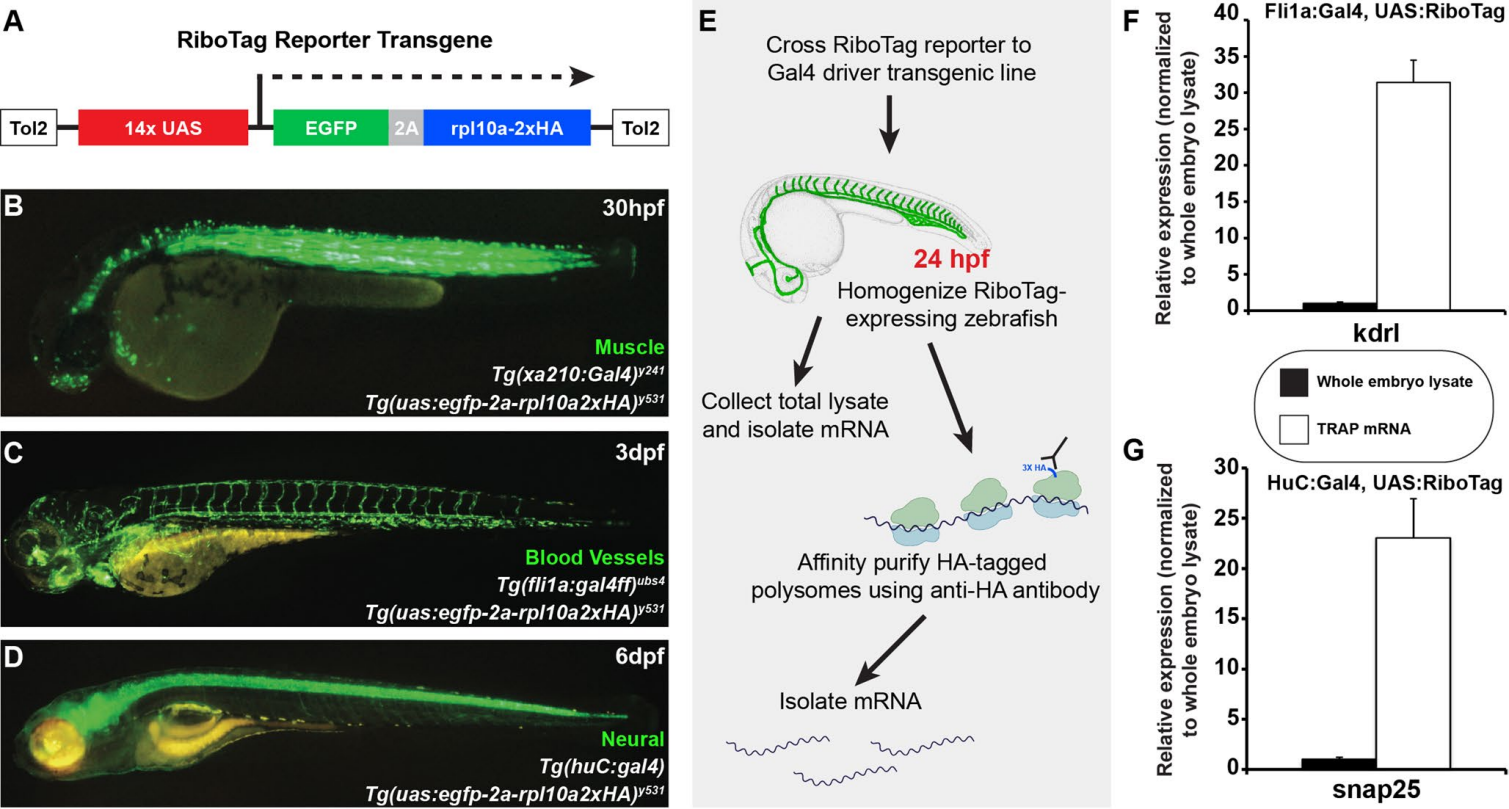
TRAP profiling can determine gene expression in different tissues and cell types using Ribotag Reporter transgenics. **A** Schematic diagram of the Tol2(uas:egfp-2a-rpl10a2xHA) RiboTag Reporter transgene. **B** Green epifluorescence photomicrograph of a 30hpf *Tg(uas:egfp-2a-rpl10a2xHA)*^*y531*^*; Tg(xa210:gal4)*^*y241*^ double transgenic animal. **C** Green epifluorescence photomicrograph of a 3dpf *Tg(uas:egfp-2a-rpl10a2xHA)*^*y531*^*; Tg(fli1a:gal4ff)*^*ubs4*^ double transgenic animal. **D** Green epifluorescence photomicrograph of a 6dpf *Tg(uas:egfp-2a-rpl10a2xHA)*^*y531*^*; Tg(huc:gal4)* double transgenic animal. **E** Schematic diagram illustrating the workflow for TRAP purification of RNAs from RiboTag Reporter zebrafish crossed to Gal4 driver lines. **F** Quantitative RT-PCR measurement of the relative expression of the endothelial-specific *kdrl* gene in samples prepared from TRAP purified RNA from *Tg(uas:egfp-2a-rpl10a2xHA)*^*y531*^*; Tg(fli1a:gal4ff)*^*ubs4*^ double-transgenic animals compared to RNA prepared whole embryo lysates from the same animals. **G** Quantitative RT-PCR measurement of the relative expression of the neural-specific *snap25* gene in samples prepared from TRAP purified RNA from *Tg(uas:egfp-2a-rpl10a2xHA)*^*y531*^*;Tg(huc:gal4)* double-transgenic animals compared to RNA prepared whole embryo lysates from the same animals

Our success in carrying out endothelial profiling using AngioTag TRAP-RNAseq led us to ask whether we could generalize this method to profile other non-endothelial tissues. To address this question, we generated a *Tg(uas: egfp-2a-rpl10a2xHA)*^*y531*^ UAS: RiboTag transgenic line to drive expression of the RiboTag cassette in any tissue or cell type of interest for which a Gal4 driver line is available (Fig. 8A). The UAS: RiboTag line showed strong, tissue-specific expression after being crossed to a variety of different Gal4 driver lines, including a muscle-specific *Tg(xa210:gal4)*^*y241*^ line [23] (Fig. 8B), an endothelial-specific *Tg(fli1a: gal4ff)*^*ubs4*^ line [24] (Fig. 8C), and a neural-specific *Tg(huC: gal4)* line [25] (Fig. 8D). TRAP isolation of mRNA from embryos obtained from each of these crosses revealed strong enrichment of tissue-specific genes in samples prepared from the appropriate lines (Fig. 8E-G), specifically *kdrl* in the endothelial line and *snap25* in the neural line. The enrichment of the endothelial-specific *kdrl* gene in TRAP-purified mRNA from *Tg(uas: egfp-2a-rpl10a2xHA)*^*y531*^; *Tg(fli1a: gal4ff)*^*ubs4*^ double-transgenic animals was comparable to that found in TRAP-purified mRNA from our *Tg(kdrl: egfp-2a-rpl10a3xHA)*^*y530*^ AngioTag line (compare Figs. 1L and 8F), suggesting that the UAS: RiboTag line provides a similarly effective tool for tissue-specific gene expression profiling.

## Discussion

In this study, we introduce a novel AngioTag transgenic line for profiling global gene expression in zebrafish vascular endothelial cells in their undisturbed endogenous environment. We have performed TRAP-RNAseq profiling of the endothelial translatome of 24 hpf AngioTag larvae, showing that TRAP-RNAseq results in superior endothelial gene enrichment compared to RNAseq on FACS-isolated endothelial cells. We have used AngioTag TRAP-RNAseq to document vascular enrichment of a number of annotated genes that were not previously known to be expressed in the endothelium, and to identify several novel unannotated vascular genes. We have also profiled the endothelial gene expression of several different adult organs, uncovering genes shared by the endothelium of all organs and genes unique to the vasculature of specific organs. Finally, to make TRAP-RNAseq methodologies accessible to all researchers and not just those studying the vascular endothelium, we have generated a UAS-RiboTag line that can be used to profile any cell and tissue type for which a Gal4 driver line is available.

Classically, translatomes have been prepared for profiling using sucrose density gradients, collecting RNA from the polysome fraction [46]. Profiling the translatome of a particular tissue type using this method requires either dissection or FACS sorting. More recently, the TRAP method has been utilized to profile the translatomes of specific tissues or cell types. With thorough validation of this technique in several model organisms demonstrating proper ribosome incorporation of tagged ribosomal subunits and positive correlations between ribosome-associated mRNA transcripts and protein expression [12, 15–20], this technique is proving to be an ideal method for tissue specific molecular profiling of cells within their in vivo environments. Using mice with a floxed conditional ‘tagged’ allele of the Rpl22 ribosomal protein expressed after crossing the mice to cell- or tissue-specific Cre driver lines [12], studies have assayed gene expression in hypothalamic neurons through TaqMan assays [47], Sertoli and Leydig cells of the testes through microarrays [20], proneural gliomas through ribosomal footprinting on translatome RNA [48], factor VII expression in endothelial cells through qPCR [49], and endothelial gene expression during homeostasis and inflammation through RNA-sequencing [50]. Other groups have utilized the TRAP method to perform RNAseq on specific cell populations in the mouse brain or kidney, with all but one group amplifying their RNA prior to sequencing [14, 16, 18, 51]. Tissue specific RiboTag zebrafish have also been generated driving expression of GFP fused to rpl10a with tissue-specific promoters and then utilizing anti-GFP antibodies to immunoprecipitate tissue-specific translatomes. Translatomes prepared in this way have been used to assay gene expression in the heart using microarrays [19] and in melanocytes using qPCR [17]. A binary zebrafish RiboTag system has also been generated in which transgenic zebrafish carrying Avi-tagged Rpl10a are crossed to tissue specific BirA lines to biotinylate Rpl10a in BirA-expressing tissues. This binary biotinylation method was used to perform microarray analysis on zebrafish skeletal muscle mRNAs [52]. Notably, the two zebrafish studies noted above using microarrays also amplified their collected translatome RNA prior to further analysis. In contrast, our larval TRAP-RNAseq procedures were performed without any amplification of collected RNA.

Our whole embryo translatome TRAP-RNAseq results provide a valuable dataset for uncovering new insights into sequence features associated with more- or less-highly translated genes. Interestingly, our data on codon usage associated with highly translated genes shows strong parallels with previously published data examining codon usage in the developing zebrafish [41]. In that study, the authors examined codon usage in the 469 most highly transcribed genes in the zebrafish genome (as determined from previously published RNAseq data), identifying codons preferentially associated with highly transcribed genes. These authors went on to show that, by replacing the native codons with codons preferred in “more transcribed” genes, they were able to increase protein accumulation, providing experimental support for the idea that zebrafish use “optimal” codons to increase translation of their already highly transcribed genes. Our data reveals that many of the same codons enriched in the highly transcribed gene set of Horstick et al. are also preferentially enriched in the most-highly translated genes in our whole-embryo translatome dataset (Fig. 3C-D), providing direct experimental evidence for the idea that highly transcribed genes are also “translationally optimized.”

Comparisons between our TRAP-RNAseq and FACS-RNAseq endothelial datasets (Fig. 4) suggest that TRAP-RNAseq yields more effective enrichment of endothelial genes. Of the top 20 enriched GO terms in each dataset, there were three times as many vascular-related biological process GO terms in the TRAP-RNAseq dataset compared to the FACS-RNAseq dataset (Fig. 4C and F). Interestingly, principal component analysis of our RNAseq data (Fig. 2D) also shows that the three AngioTag TRAP-RNAseq replicates (red spheres) are more highly clustered with one another and with the other non-FACS RNAseq datasets (blue, magenta, and gold spheres) than the RNAseq data from either FACS-sorted endothelial cells (green spheres) or from dissociated but unsorted total embryonic cells (teal spheres), which are much less clustered with the other RNAseq datasets. These results suggest that technical aspects of the FACS process, such as embryonic dissociation and prolonged incubation of separated cells prior to collection of mRNA (samples 3 and 4 in Fig. 2C), introduce significant changes in gene expression compared to samples in which cells are rapidly disrupted prior to either immediate collection of mRNA (samples 1 and 5 in Fig. 2C) or TRAP-RNAseq (samples 2 and 6 in Fig. 2C). This idea is further supported by the strong up-regulation of stress induced genes observed in the dissociated samples (Supplemental Fig. 1). Using our TRAP-RNAseq methods, we were able to identify previously uncharacterized endothelial genes. These include both annotated genes not previously shown to have endothelial-enriched expression (Fig. 5A–E) and unannotated genes (Fig. 5F–O), some of which we have also shown are required for normal vascular development (Fig. 6). These results further reinforce the idea that the AngioTag TRAP-RNAseq approach can provide unique insights compared to FACS sorting of dissociated endothelial cells.

We also show that AngioTag TRAP-RNAseq provides an effective method for querying and comparing the endothelial translatomes of different adult organs. Comparing the endothelial translatome of each organ to its own tissue lysate reveals shared endothelial signatures across all organs (Fig. 7B). Pairwise comparisons between the endothelial translatomes of each organ also reveals unique, organ-specific endothelial profiles (Fig. 7I), uncovering diversity among the vascular beds of different organs that likely reflect functional differences between the vessels found in different tissues. These findings further reinforce the importance of profiling the vasculature within its native in vivo environment since it is evident that tissue location plays an important role in vascular gene expression.

Finally, the new *Tg(uas: egfp-2a-rpl10a2xHA)*^*y531*^ UAS: RiboTag line that we have generated in the course of this work makes it possible to apply the TRAP-RNAseq methods we used to profile the endothelium to any cell or tissue type in the zebrafish for which a Gal4 driver line is available (Fig. 8). A very large number of transgenic Gal4 driver lines have been generated in the zebrafish with expression in a wide assortment of different cells and tissues, including many lines derived from enhancer trap screens [53–56]. The availability of these lines should allow the wide application of our TRAP-RNAseq methods.

Together, the new tools and methods we have developed for transgene-assisted TRAP-RNAseq provide a valuable resource for cell- and tissue-specific in vivo expression profiling in developing and adult zebrafish. By combining these new TRAP-RNAseq profiling tools with other genetic and experimental tools available in the zebrafish, we can study changes in gene expression due to gene mutations, disease states, or during regeneration in numerous cell types. These new tools and methods greatly enhance our capability to analyze changes in translational landscapes within cells in their local microenvironments.

## Electronic supplementary material

Below is the link to the electronic supplementary material.

Supplementary Material 1

Supplementary Material 2

Supplementary Material 3

Supplementary Material 4

Supplementary Material 5

Supplementary Material 6

Supplementary Material 7
