# Supplementary material for: In vivo profiling of the endothelium using ‘AngioTag’ zebrafish
Source: Angiogenesis. 2025 Jul 4;28(3):40. doi: 10.1007/s10456-025-09990-8 (PMC12227374; doi:10.1007/s10456-025-09990-8)
Supplement: Supplementary file 1 — Supplementary Material 1 [file 10456_2025_9990_MOESM1_ESM.docx]

**SUPPLEMENTAL DATA**

**Supplemental Figures**


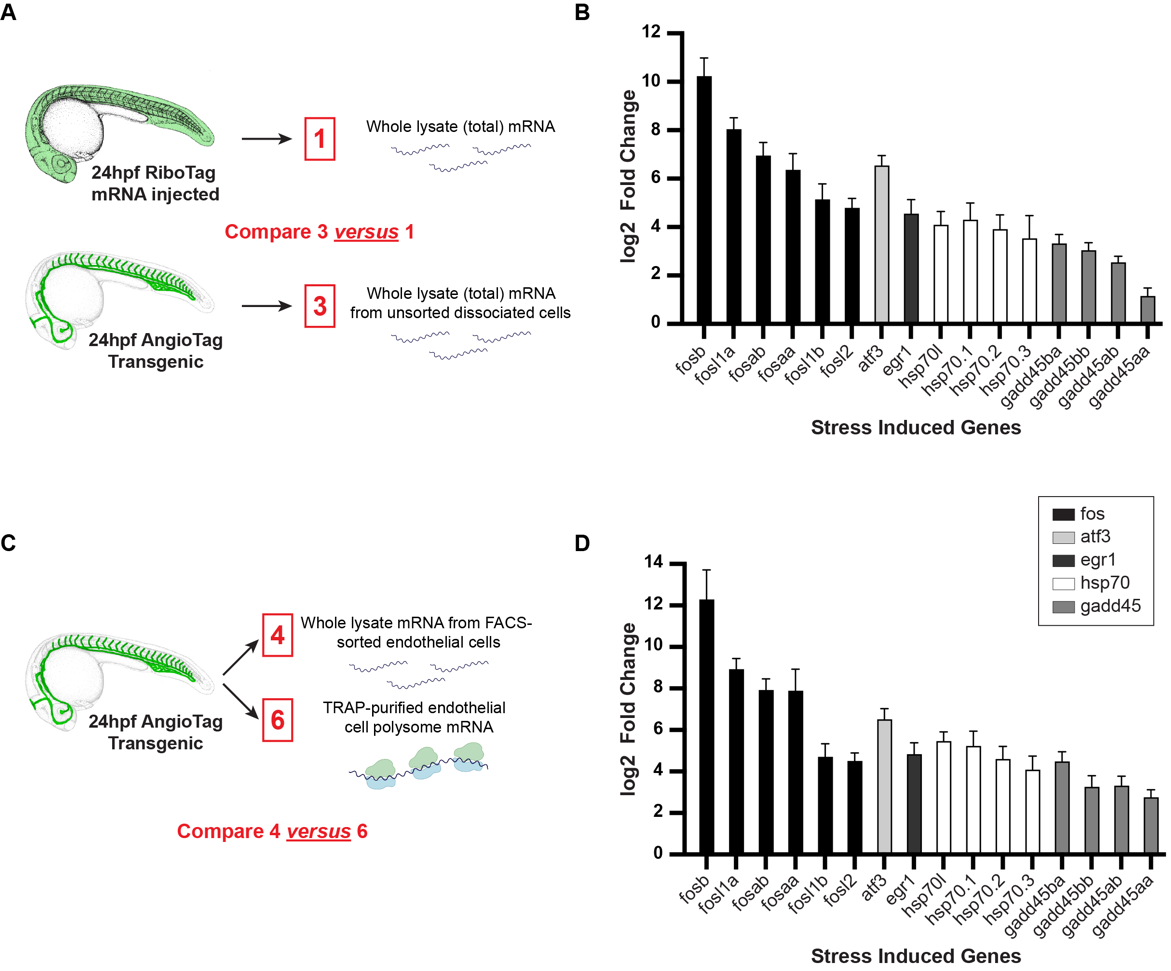


**Supplemental Figure 1. Cell dissociation leads to increased stress-induced gene expression**

**(A)** Schematic depicting samples collected for RNAseq analysis of whole animal transcriptome from unsorted dissociated cells (sample 3) compared to whole embryo lysate (sample 1). **(B)** Bar graph showing the log2 fold enrichment of stress induced genes from the comparison depicted in panel A. Colored bars indicate different gene families. **(C)** Schematic depicting samples collected for RNAseq analysis of endothelial gene expression from FACs-sorted endothelial cells (sample 4) compared to TRAP-purified endothelial cell polysome mRNA (sample 6). **(D)** Bar graph showing the log2 fold enrichment of stress induced genes from the comparison depicted in panel C. Colored bars indicate different gene families.

**Supplemental Tables**

Supplemental Table 1: Readcounts for larval RNAseq analysis

| **Sample** | **Total mapped reads** | **Uniquely**  **mapped reads** | **% uniquely**  **mapped reads** | **Reads mapped to multiple loci** | **% reads mapped to multiple loci** |
| --- | --- | --- | --- | --- | --- |
| 1-1 RiboTag Whole Lysate | 49956134 | 42681474 | 85.44% | 3043134 | 6.09% |
| 1-2 RiboTag Whole Lysate | 69239038 | 59468880 | 85.89% | 3119373 | 4.51% |
| 1-3 RiboTag Whole Lysate | 47632455 | 41805225 | 87.77% | 2030482 | 4.26% |
| 2-1 RiboTag TRAP | 50661654 | 35008681 | 69.10% | 3826393 | 7.55% |
| 2-2 RiboTag TRAP | 56252261 | 43029188 | 76.49% | 4023465 | 7.15% |
| 2-3 RiboTag TRAP | 45403187 | 36469445 | 80.32% | 2409121 | 5.31% |
| 3-1 AngioTag FACS Total Cells | 55085749 | 51701925 | 93.86% | 1598792 | 2.90% |
| 3-2 AngioTag FACS Total Cells | 53707170 | 49412362 | 92.00% | 1452744 | 2.70% |
| 3-2 AngioTag FACS Total Cells | 58235304 | 51473076 | 88.39% | 2112884 | 3.63% |
| 4-1 AngioTag FACS ECs | 56759660 | 50935960 | 89.74% | 1402901 | 2.47% |
| 4-2 AngioTag FACS ECs | 55032277 | 29503831 | 53.61% | 21559807 | 39.18% |
| 4-3 AngioTag FACS ECs | 52123159 | 45771455 | 87.81% | 2585146 | 4.96% |
| 5-1 AngioTag Whole Lysate | 46722553 | 40348948 | 86.36% | 1982780 | 4.24% |
| 5-2 AngioTag Whole Lysate | 57354200 | 54080083 | 94.29% | 1894448 | 3.30% |
| 5-3 AngioTag Whole Lysate | 53618024 | 50363143 | 93.93% | 1890768 | 3.53% |
| 6-1 AngioTag TRAP | 49463459 | 26669161 | 53.92% | 7878773 | 15.93% |
| 6-2 AngioTag TRAP | 58760419 | 55094356 | 93.76% | 2018623 | 3.44% |
| 6-3 AngioTag TRAP | 55289491 | 51421138 | 93.00% | 2200667 | 3.98% |

Supplemental Table 2: Top 10 GO terms for skin AngioTag

| **Skin AngioTag:**  **GO Biological Process** | **Number of genes in set** | **Mean log2fold change** | **Adjusted**  **p-value** |
| --- | --- | --- | --- |
| angiogenesis (GO:0001525) | 129 | 1.80 | 2.33E-08 |
| actin filament organization (GO:0007015) | 127 | 1.73 | 6.32E-09 |
| blood vessel morphogenesis (GO:0048514) | 163 | 1.72 | 6.72E-12 |
| small GTPase mediated signal transduction (GO:0007264) | 129 | 1.58 | 6.72E-12 |
| regulation of anatomical structure morphogenesis (GO:0022603) | 136 | 1.58 | 1.31E-09 |
| regulation of small GTPase mediated signal transduction (GO:0051056) | 61 | 1.53 | 1.25E-06 |
| positive regulation of transcription by RNA polymerase II (GO:0045944) | 118 | 1.50 | 1.24E-07 |
| regulation of GTPase activity (GO:0043087) | 87 | 1.47 | 1.61E-09 |
| chromatin organization (GO:0006325) | 116 | 1.41 | 2.33E-08 |
| RNA splicing (GO:0008380) | 112 | 1.31 | 1.32E-06 |

Supplemental Table 3: Top 10 GO terms for muscle AngioTag

| **Muscle AngioTag:**  **GO Biological Process** | **Number of genes in set** | **Mean log2fold change** | **Adjusted**  **p-value** |
| --- | --- | --- | --- |
| angiogenesis (GO:0001525) | 153 | 2.41 | 4.62E-20 |
| blood vessel morphogenesis (GO:0048514) | 183 | 2.33 | 1.47E-22 |
| chromatin organization (GO:0006325) | 127 | 1.58 | 1.13E-14 |
| histone modification (GO:0016570) | 102 | 1.51 | 4.58E-13 |
| regulation of mRNA metabolic process (GO:1903311) | 78 | 1.50 | 3.00E-12 |
| RNA splicing (GO:0008380) | 143 | 1.42 | 8.26E-21 |
| mRNA processing (GO:0006397) | 162 | 1.42 | 2.29E-20 |
| mRNA splicing, via spliceosome (GO:0000398) | 115 | 1.40 | 1.64E-19 |
| RNA splicing, via transesterification reactions (GO:0000375) | 115 | 1.40 | 1.64E-19 |
| RNA splicing via transeterfication reaction with bluged adenosine as nucleophile (GO:0000377) | 115 | 1.40 | 1.64E-19 |

Supplemental Table 4: Top 10 GO terms for liver AngioTag

| **Liver AngioTag:**  **GO Biological Process** | **Number of genes in set** | **Mean log2fold change** | **Adjusted**  **p-value** |
| --- | --- | --- | --- |
| lymphangiogenesis (GO:0001946) | 25 | 3.69 | 9.73E-09 |
| lymph vessel morphogenesis (GO:0036303) | 26 | 3.67 | 1.09E-89 |
| lymph vessel development (GO:0001945) | 35 | 3.36 | 1.07E-10 |
| sprouting angiogenesis (GO:0002040) | 49 | 3.20 | 1.81E-08 |
| angiogenesis (GO:0001525) | 124 | 3.00 | 4.66E-22 |
| blood vessel morphogenesis (GO:0048514) | 148 | 2.96 | 2.48E-25 |
| regulation of anatomical structure morphogenesis (GO:0022603) | 97 | 2.72 | 3.31E-09 |
| ameboidal-type cell migration (GO:0001667) | 79 | 2.65 | 1.94E-09 |
| actin filament organization (GO:0007015) | 93 | 2.51 | 2.89E-09 |
| small GTPase mediated signal transduction (GO:0007264) | 98 | 2.50 | 3.06E-13 |

Supplemental Table 5: Top 10 GO terms for heart AngioTag

| **Heart AngioTag:**  **GO Biological Process** | **Number of genes in set** | **Mean log2fold change** | **Adjusted**  **p-value** |
| --- | --- | --- | --- |
| actin filament organization (GO:0007015) | 86 | 1.69 | 1.76-09 |
| angiogenesis (GO:0001525) | 101 | 1.68 | 1.41E-14 |
| blood vessel morphogenesis (GO:0048514) | 120 | 1.64 | 2.14E-16 |
| stem cell differentiation (GO:0048863) | 70 | 1.59 | 5.32E-08 |
| embryonic organ morphogenesis (GO:0048562) | 103 | 1.58 | 2.67E-09 |
| regulation of multicellular organismal development (GO:2000026) | 95 | 1.58 | 4.50E-08 |
| small GTPase mediated signal transduction (GO:0007264) | 78 | 1.56 | 4.50E-08 |
| regulation of anatomical structure morphogenesis (GO:0022603) | 91 | 1.53 | 9.55E-10 |
| skeletal system development (GO:0001501) | 93 | 1.53 | 1.76E-09 |
| chromatin organization (GO:0006325) | 84 | 1.52 | 9.32E-11 |

Supplemental Table 6: Top 10 GO terms for brain AngioTag

| **Brain AngioTag:**  **GO Biological Process** | **Number of genes in set** | **Mean log2fold change** | **Adjusted**  **p-value** |
| --- | --- | --- | --- |
| sprouting angiogenesis (GO:0002040) | 47 | 3.28 | 3.20-05 |
| angiogenesis (GO:0001525) | 113 | 3.23 | 1.18E-11 |
| blood vessel morphogenesis (GO:0048514) | 137 | 3.18 | 5.34E-14 |
| regulation of anatomical structure morphogenesis (GO:0022603) | 95 | 2.49 | 3.20E-05 |
| regulation of cells shape (GO:0008360) | 34 | 2.47 | 8.55E-06 |
| Ras protein signal transduction (GO:0007265) | 70 | 2.45 | 7.48E-07 |
| small GTPase mediated signal transduction (GO:0007264) | 95 | 2.35 | 4.27E-08 |
| regulation of hydrolase activity (GO:0051336) | 99 | 2.34 | 3.20E-05 |
| regulation of cell morphogenesis (GO:0022604) | 41 | 2.31 | 9.83E-07 |
| regulation of cysteine-type endopeptidase activity involved in apoptotic process (GO:0043281) | 23 | 2.28 | 3.20E-05 |
